# Plate tectonics drive tropical reef biodiversity dynamics

**DOI:** 10.1038/ncomms11461

**Published:** 2016-05-06

**Authors:** Fabien Leprieur, Patrice Descombes, Théo Gaboriau, Peter F. Cowman, Valeriano Parravicini, Michel Kulbicki, Carlos J. Melián, Charles N. de Santana, Christian Heine, David Mouillot, David R. Bellwood, Loïc Pellissier

**Affiliations:** 1 UMR MARBEC, (CNRS, IRD, IFREMER, UM), cc 093, Place E. Bataillon, FR-34095 Montpellier, France; 2 University of Fribourg, Unit of Ecology & Evolution, Ch. du Musée 10, CH-1700 Fribourg, Switzerland; 3 Swiss Federal Research Institute WSL, CH-8903 Birmensdorf, Switzerland; 4 Landscape Ecology, Institute of Terrestrial Ecosystems, ETH Zürich, 8044 Zürich, Switzerland; 5 Department of Ecology & Evolutionary Biology, Yale University, 21 Sachem St, New Haven, Connecticut 16 06511, USA; 6 CRIOBE, USR 3278 CNRS-EPHE-UPVD, LABEX ‘CORAIL', University of Perpignan, 66860 Perpignan, France; 7 CESAB-FRB, Immeuble Henri Poincaré, Domaine du Petit Arbois, FR-13857 Aix-en-Provence, France; 8 Institut pour la Recherche en Développement, UR UMR "Entropie" -Labex Corail- Université de Perpignan, 66000 Perpignan, France; 9 Department of Fish Ecology and Evolution, Eawag: Swiss Federal Institute of Aquatic Science and Technology 6047 Kastanienbaum, Switzerland; 10 EarthByte Group, The University of Sydney, Sydney, New South Wales 2006, Australia; 11 Australian Research Council Centre of Excellence for Coral Reef Studies, James Cook University, Townsville, Queensland 4811, Australia; 12 College of Marine and Environmental Sciences, James Cook University, Townsville, Queensland 4811, Australia

## Abstract

The Cretaceous breakup of Gondwana strongly modified the global distribution of shallow tropical seas reshaping the geographic configuration of marine basins. However, the links between tropical reef availability, plate tectonic processes and marine biodiversity distribution patterns are still unknown. Here, we show that a spatial diversification model constrained by absolute plate motions for the past 140 million years predicts the emergence and movement of diversity hotspots on tropical reefs. The spatial dynamics of tropical reefs explains marine fauna diversification in the Tethyan Ocean during the Cretaceous and early Cenozoic, and identifies an eastward movement of ancestral marine lineages towards the Indo-Australian Archipelago in the Miocene. A mechanistic model based only on habitat-driven diversification and dispersal yields realistic predictions of current biodiversity patterns for both corals and fishes. As in terrestrial systems, we demonstrate that plate tectonics played a major role in driving tropical marine shallow reef biodiversity dynamics.

## Abstract (web summary)

Over a geological timescale, plate tectonics are thought to promote biodiversity, but this link remained descriptive. Here, Leprieur *et al*. model dynamically how plate tectonics shaped species diversification and movement of hotspots on tropical reefs over the past 140 million years.

## Graphical abstract

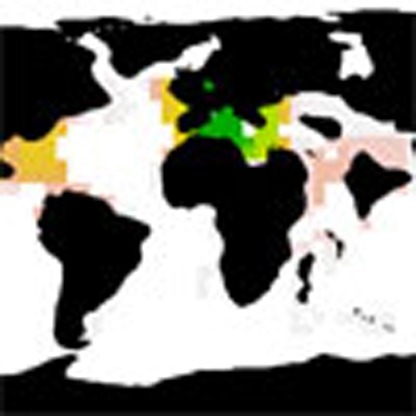

Tropical shallow reefs support the world's greatest diversity of marine organisms^1^ and have experienced a long history of climate-driven habitat change^2^,^3^. Sea level and temperature fluctuations during the Quaternary have strongly shaped the distribution of coral reefs and their associated fish faunas^3^. Nevertheless, most of the diversification of tropical marine faunas predates the Quaternary^4^, arguing for a major role of older historical events^2^,^4^,^5^. Because plate tectonics modulate the distribution of seafloors through continental movement and collision over geological timescales^6^, they are potentially one of the main determinants of tropical marine biodiversity^2^,^5^. The fossil record indicates that the location of the major marine biodiversity hotspot has moved across the globe during the last 50 million years^2^, transitioning from the western Tethys during the Eocene (55–33 Ma), to the Arabian Peninsula and Western Indian Ocean during the Late Eocene and Oligocene (37–15 Ma), and finally to the Indo-Australian Archipelago (IAA) in the Miocene (15–5 Ma). This movement was primarily driven by successive closure of the Tethys followed by the collision of the Arabian and Indian plates with Eurasia. Yet, the role of plate tectonics in shaping diversification and dispersal processes of tropical marine biodiversity still remains elusive. Correlative approaches have had limited success in disentangling alternative hypotheses and mechanistic models may provide a clearer understanding of the processes shaping marine biogeographic patterns^7^,^8^.

We coupled a mechanistic model of species diversification to a model of synthetic paleobathymetry estimates for the past 140 Myr. The gridded paleobathymetry model is based on global ocean paleo-age grids^6^ merged with paleocoastline distributions for continental areas^9^. The model outlines the absolute position of continents over time and the associated distribution and residence time of shallow epicontinental seas. Using a novel dynamic model that simulates clade dispersal, speciation and extinction from a single ancestral species, bounded by a temporal sequence of habitat availability, we show that plate motions predict the emergence and movement of diversity hotspots on tropical reefs. Incipient species emerge within (that is, sympatric) or adjacent (that is, parapatric) to the geographical range of its ancestor^10^,^11^ and disperse at a distance *d* during each time step. Parapatric speciation arises when a species range is split into one or more distinct areas separated by a distance *d*_s_, while sympatric speciation arises within a cell with a probability *P*_s_. Consistent with the speciation-area hypothesis^12^, species occupying a large area show higher sympatric speciation frequency *S=P*_s_ × *A*. Regardless of the speciation mode, the models predict the emergence and movement from the western Tethys towards the IAA. The diversification model assumes that speciation and extinction are modulated exclusively by variations in habitat size and spatial distribution. The model is based on strict niche conservatism, but provides a realistic picture of the historical dynamic of marine reef fauna.

## Results and Discussion

### Hopping biodiversity hotspots

**Figure 1:**
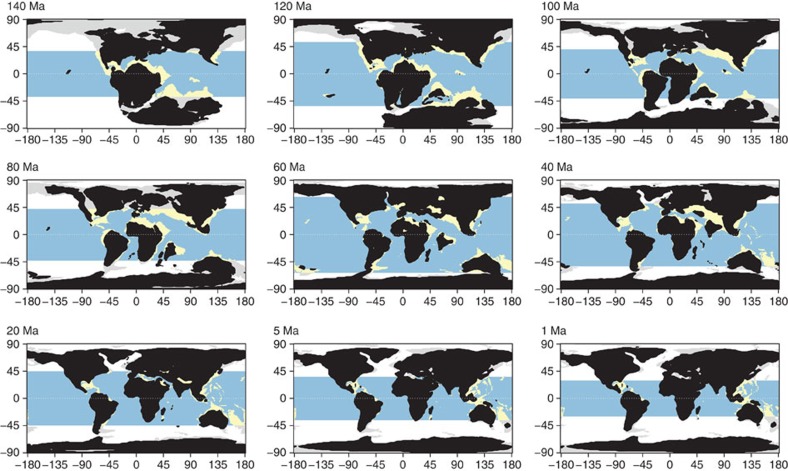
Distribution of shallow and deep ocean sea floor across the past 140 Myr. The latitudinal tropical limit was obtained from the fossil distribution of coral species. Light blue represents deep tropical ocean, while yellow represents tropical shallow reefs. White and light grey represent deep ocean and shallow waters outside the tropical boundary, respectively.

**Figure 2:**
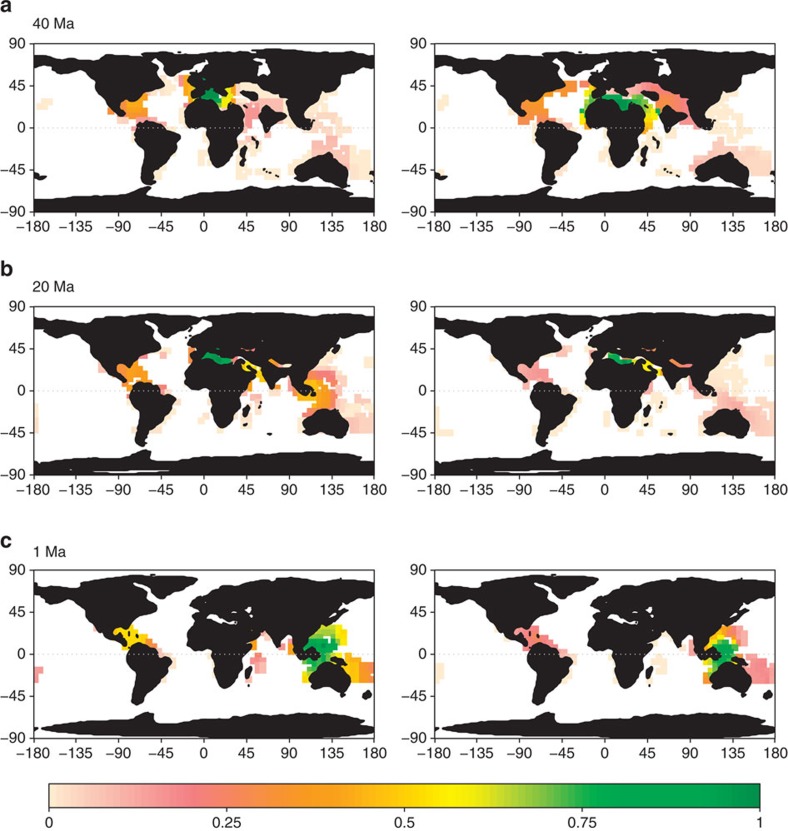
Hopping biodiversity hotspots. Shown are the hopping hotspot observed from coral fossils (left) and simulated with the parapatric model (right) for three time periods, Eocene (**a**), Miocene (**b**) and Quaternary/current (**c**). The two most ancient time periods depict observed diversity interpolated from coral fossil records (www.paleodb.org), while the most recent period shows current coral diversity (IUCN). Diversity values were rescaled between 0 (minimum, pink) and 1 (maximum, green). The best model provided good correlation with Eocene and Miocene (*d*=4, *d*_s_=5, 40 Ma: *R*^2^=0.26; 20 Ma: *R*^2^=0.38) and with present-day diversity (*d*=4, *d*_s_=5, fish: *R*^2^=0.36; coral: *R*^2^=0.42).

We show that the spatial distribution of tropical reef habitats over time (Fig. 1; Supplementary Fig. 1) explains the emergence and movement of coral reef biodiversity hotspots since the Cretaceous with both parapatric (Supplementary Fig. 2) and sympatric (Supplementary Fig. 3) speciation modes (Fig. 2; Supplementary Fig. 4). The parapatric model (*d=*4, *d*_s_=5) provided better estimates of fossil biodiversity in the Eocene (40 Ma, *R*^2^=0.26) and the Miocene (20 Ma, *R*^2^=0.38) than the sympatric model (*d*=5, *P*_s_=6e−5, 40 Ma: *R*^2^=0.12; 20 Ma: *R*^2^=0.13). The paleobathymetry model suggests that the Tethys contained extensive but disjointed reef habitats promoting both modes of speciation (Supplementary Fig. 5), but the biodiversity peak in the Western Tethys is most consistent with parapatric speciation fuelled by waves of disconnection and reconnection of isolated reef regions (Supplementary Fig. 6). The highly dynamic and complex tectonics of the Western Tethys^13^ provided fertile ground for parapatric speciation. Modelled diversification, computed as speciation minus extinction rates, peaked at 85 Ma (Supplementary Figs 7 and 8) when fragmentation was highest, underpinning the biodiversity hotspot in Western Tethys (Supplementary Fig. 5). During the mid/late Eocene–Oligocene times, the closure of the Tethys was associated with a narrowing coastal corridor^14^, that allowed Tethyan biodiversity to move eastward (Fig. 2), supporting the hypothesis of faunal migration into the IAA^2^,^15^. Since high faunal turnover is expected at the transition across the Cretaceous–Paleogene (K/Pg)^16^,^17^, which is not considered in our habitat reconstructions, a sensitivity analyses demonstrated the robustness of this dynamic to a high degree of forced extinction at 66 Ma (80%, Supplementary Fig. 9). In addition, our results showed robustness in regards to the choice of plate kinematic model used to reconstruct plate positions (Supplementary Fig. 10). Together, these results indicate that the Tethys was a major diversification and emigration hotspot explaining the Tethyan origin of most tropical shallow reef lineages, here shown for corals, but with parallels in fishes^18^ and foraminifera^2^.

### Current biodiversity patterns

We examined whether the parapatric and sympatric models predicted realistic current biodiversity patterns for two globally distributed coral (Acroporidae) and fish (Labridae) clades. Parapatric (*R*^2^=0.36 and *R*^2^=0.42) and sympatric *R*^2^=0.46 and *R*^2^=0.45) models both explain global variation in coral and fish species richness, which peaks in the Central Indo-Pacific region, and more particularly in the IAA, centred between Indonesia and Papua New Guinea (Fig. 2; Supplementary Fig. 4). The lower diversity in the Atlantic is not only the result of less extensive Quaternary refugia^3^, but also lower opportunity for immigration and speciation since the Eocene than in the geologically complex Central Indo-Pacific region^18^,^19^ (Supplementary Fig. 5). The models also provided realistic values of between-assemblage (*β*) diversity for both parapatric (Acroporidae: mantel *r*_m_=0.56; Labridae: *r*_m_=0.63) and sympatric (Acroporidae: *r*_m_=0.76; Labridae: *r*_m_=0.77) speciation modes. Observed and predicted values of assemblage dissimilarity increase with sea distance, and the model captures the high level of faunal turnover between the Atlantic and Indo-Pacific oceans^20^ (Supplementary Fig. 11), which were largely disconnected after the closure of the Tethys Sea^18^. Overall, these results demonstrate the important role played by isolation and plate tectonics in shaping patterns of speciation and dispersal, and in explaining the present-day longitudinal gradient in tropical marine shallow reef biodiversity.

### Nestedness patterns and the merging of lineages

**Figure 3:**
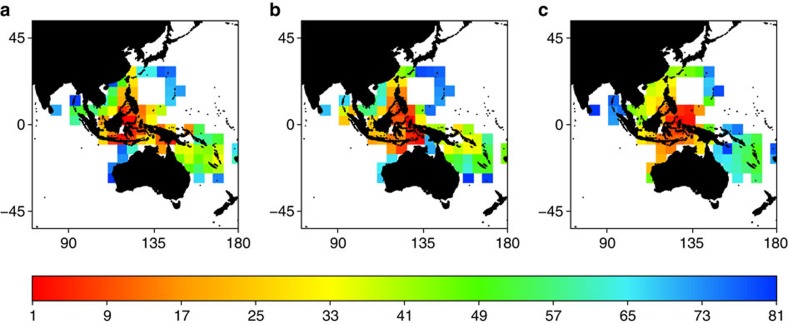
Nestedness pattern in the Central Indo-Pacific region. Observed (**a**,**b**) and simulated (**c**) pattern of nestedness in assemblage shown for (**a**) fishes and (**b**) corals and (**c**) with the parapatric model (*d*=4, *d*_s_=5) in the Central Indo-Pacific region. The colour scale represents the rank order of cells from the most species-rich cells (red) to the less species-rich ones (blue). The parapatric model faithfully outlines the current pattern of nestedness across the Central Indo-Pacific region, namely that assemblages in peripheral and species-poor cells are composed of species that constitute subsets of species that occur in successively richer cells of the Indo-Australian Archipelago (IAA).

**Figure 4:**
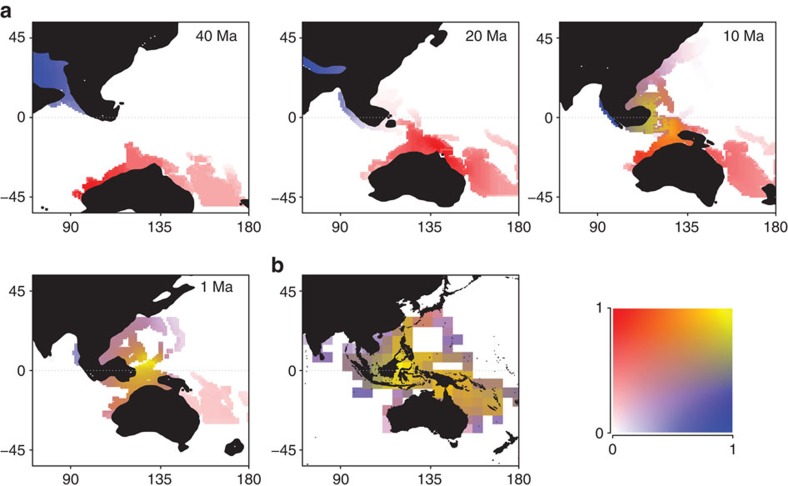
Biodiversity patterns through time. (**a**) Shown are results of the parapatric model (*d*=4, *d*_s_=5) for the Tethyan (blue) and Australian (red) lineages in the Central Indo-Pacific (CIP) region for 40, 20, 10 and 1 Myr ago. The merging of lineages is represented by the yellow colour. (**b**) Current richness of the Tethyan (parrotfish, scarines, in blue) and Australian (hypsigenyines and pseudocheilines in red) lineages. The ancestral origins were inferred from a biogeographic reconstruction based on a DEC model (Supplementary Fig. 14). The distribution of these two labrid lineages may still show a relict signal of those origins, with a high proportion of hypsigenyines and pseudocheilines along the coast of Australia.

The parapatric model accurately (Acroporidae, Spearman' rank correlation: *r*_s_=0.54, *P*<0.001, Labridae, *r*_s_=0.59, *P*<0.001) mimicked the nested structure of assemblages in the Central Indo-Pacific region^19^,^21^ (Parapatric model: NODF_sites_=33.2, *P*<0.001; fish: NODF_sites_=82.9, *P*<0.001; coral: NODF_sites_=90.5, *P*<0.001; Fig. 3). Assemblages in peripheral and species-poor cells are composed of species that constitute subsets of species that occur in successively richer cells of the IAA (Fig. 3). Furthermore, these peripheral areas display distinct tropical reef faunas, which are also mimicked by our mechanistic model (Supplementary Fig. 12). The mechanistic model predicts that two faunas previously separated, from Tethys and coastal Australia, fused when the Sunda and Sahul shelves entered in collision (Fig. 4; Supplementary Fig. 13). Among the candidate lineages of Australian origin, a biogeographic reconstruction based on the labrid phylogeny (Supplementary Fig. 14) suggests that the hypsigenyine and pseudocheiline lineages became isolated in the Central Indo-Pacific region in the Eocene before the emergence of the IAA. Those may have colonized the Australian coast across the Southern Indian Ocean (Supplementary Fig. 15). In contrast, the scarines, a lineage with earliest fossils in Tethys, represents a more recent colonization in the Miocene from Tethys (Supplementary Fig. 15). This, together with other evidence for sharks^22^ and corals^23^ may suggest the existence of an Australian endemic marine fauna before the Miocene. The confluence of two major faunal elements, Tethyan and Australian (Fig. 4; Supplementary Fig. 14) shaped the accumulation of biodiversity in the IAA^18^, which provides a marine analogue to the juxtaposed terrestrial taxa across the Wallace line^24^.

Paralleling major radiations in terrestrial systems associated to island formation^25^ or orogeny^26^, our study highlights how the dispersal and amalgamation of continental plates since the Cretaceous has shaped the dynamics of biodiversity for two major groups of tropical marine organisms, namely corals and fishes. Both speciation modes emphasize that diversification of shallow reef biodiversity and its movement across the globe was contingent on habitat configuration (Fig. 2; Supplementary Fig. 2). Our model corroborating the nestedness pattern of assemblages suggests that the marine fauna in IAA was formed, at least in part, by the merging of Australian and Tethyan faunas. Yet, determining the exact origin of extant lineages will remain challenging given the potential for long distance dispersal in the marine realm, which, together with extinction, may blur phylogeographical inferences^18^. Together, our next-generation dynamic macroecological model provides new hypotheses with which to explore and to interpret empirical patterns, such as the fusion of the Tethyan and Australian faunas in the IAA (Figs 3 and 4). While our model assumes niche conservatism, ecological diversification is also expected to have driven diversification on tropical^11^,^27^ but bounded by available tropical reef habitat through time. Incorporating ecological niche evolution together with habitat changes into a mechanistic model of species diversification would be a new avenue of research for understanding tropical marine biodiversity.

Our results remain contingent on the quality of the synthetic paleobathymetry, reconstructed from multiple data sources with their own uncertainty, propagating up from simple navigational errors for the marine surveys collecting magnetic anomaly data^6^ to larger spatio-temporal uncertainties associated to the location of paleo-shorelines. We used two absolute plate kinematic models based on absolute moving hotspot reference frames, one of them corrected for the effects of true polar wander (TPW) before 100 Ma (ref. 28). TPW is an effect that causes the whole mantle and lithosphere to rotate latitudinally with respect to the Earth's spin axis, commonly only detectable by paleomagnetic reference frames and not visible in mantle/hotspot reference frames^29^. However, the magnitude of TPW over the past 140 Ma is debated^29^. Our two input plate kinematic models result in identical paleolatitudinal positions for a hypothetical point (20° E 33° N) on the northern edge of Africa for the time from 100 Ma to the present (Supplementary Fig. 16), where most of the species diversification occurred (Supplementary Fig. 7). Comparison with a pure paleomagnetic model^29^ shows that there are up to 700–800 km (at 60 Ma) of potential paleolatitudinal difference in the location of this point between our models and the paleomagnetic model (Supplementary Fig. 16). The habitat complexity shaping reef biodiversity would still remain in the tropical climate zones even considering a paleomagnetic reference frame and our results are robust to this uncertainty. Moreover, our models are corroborated by fossil records, indicating a prolonged state of tropical climatic conditions associated to habitat complexity in the Western Tethys at the end of Cretaceous to Eocene, fuelling species diversification^2^,^30^.

Here, we highlight the major role of plate tectonics in shaping biodiversity patterns in the tropical marine realm, but we acknowledge that other shifts in oceanic conditions such as episodic high temperature, acidity levels^31^, or ecological conditions such as habitat complexity^32^ have also influenced biodiversity dynamics. In addition, even though our process-based model provides realistic patterns of tropical marine biodiversity dynamics, this does necessarily mean that the process is realistic, arguing for further validation. Overall, our study paves the way toward a new generation of spatially explicit diversification models^8^, integrating paleo-environmental changes, providing a potential mechanistic understanding of extant biodiversity patterns.

## Methods

### Bathymetry layers and tropical limit across time

Plate tectonics drive the evolution of oceanic basins and mountain chains through the motions of continental plates across the surface of the Earth^6^. Plate kinematic models provide insights on whether and when plates are located in favourable latitudes for the establishment of tropical marine biodiversity associated with shallow water reef systems. We used two different absolute plate motion models extending back to 140 Ma, constructed using relative plate motions derived from current marine magnetic anomalies and oceanic fracture zones^33^. Different choices of reference frames determine the absolute positions of plates back in time for each model. The first model (Model 1), is based on a combined moving Indian/Atlantic Hotspot reference frame^34^ back until 100 Ma, and a fixed hotspot reference frame^35^ back to 140 Ma. For the Pacific Plate, we used the absolute reference frame from Wessel *et al*.^36^ The second model (Model 2) uses a plate kinematic framework published by Seton *et al*.^37^ This second model uses a hybrid reference frame, which merges a moving Indian/Atlantic hotspot reference frame back to 100 Ma (ref. 34) with a paleomagnetically derived true polar wander corrected reference frame^38^ back to 140 Ma.

For subducted ocean crust, the marine magnetic anomalies of *in situ* oceanic crustal remnants are utilized in combination with geological information and paleomagnetic data from accreted terranes and microcontinents along with the rules of plate tectonics to generate sets of synthetic paleo-age grids for oceanic lithosphere for the past 140 Myr. Due to the age-depth relationship related to the cooling of oceanic crust, one can compute the paleobathymetry of oceanic crust, assuming either no sedimentation or relatively simple sedimentation patterns based on present-day observations^6^. We used the GDH-1 cooling model^39^ to compute the paleobathymetry grids for each time step. Estimates for continental topography, including continental margins, however, do not follow such relatively simple rules due to the effects of tectonic deformation, sediment loading and erosion. We used a set of newly digitized paleo-shorelines^9^ from the compilation of Smith *et al*.^40^ to generate simple continental shelf topography fringing continental plates at each reconstruction time step. For both models, the depth at the continent–ocean boundary is determined by age of the adjacent oceanic crust. We combine both data sets to generate synthetic paleobathymetry models with a 1° spatial resolution for the past 140 Myr in 1 million year time steps. Shelfal area are assigned a constant bathymetry of −200 m in the grids, but this represents a coarse depth approximation of the entire shelfal area which in reality displays a much more complex bathymetry. The bathymetric reconstructions only partially considers volcanic activity leading to shelf emergence and we did not model the biodiversity of the Tropical Eastern Pacific that contains many islands of volcanic origin.

Since the study focuses on the history of tropical shallow reef fauna and because climate strongly fluctuated in the last 140 millions of years^41^, we also reconstructed the paleo-latitudes of tropical ocean limits from reef-forming coral fossil records. Reef-forming coral species can only develop at warm water temperature >25 °C (ref. 42) and thus the paleo-distribution of this group constitutes a good indicator of the past latitudinal tropical borders. We collected fossil occurrences of scleractinian corals from paleoDB ( www.paleodb.org) corresponding to 31,392 occurrences^43^ from −140 Ma to the present (Supplementary Fig. 17). From the reconstructed paleo-latitude for each million year time slice, we computed the 95th percentile of the paleo-latitude at which corals were living to inform on the latitudinal border of tropical oceans across time. By combining reconstructed shelfal areas with tropical limits, we generated 1 map per million years of tropical shallow marine habitats for the last 140 Myr (Supplementary Fig. 1).

### Mapping current and past biodiversity patterns

Current biodiversity patterns were extracted from a global-scale distribution database^3^,^44^ for tropical reef fishes and from the IUCN (http://www.iucnredlist.org/technical-documents/spatial-data) for Scleractinian corals. By examining almost 500 references and extracting information from published works, regional checklists, monographs on specific families or genera, and reports, we obtained information on presence/absence for 6,316 reef fishes in grid cells of 5° × 5°, corresponding to ∼555 × 555 km at the Equator. To compare model simulations and observed biodiversity gradients, we focused on one coral clade and one fish clade based on two criteria: the clade should show a global distribution and have a stem age within the time frame of the simulations. For the fishes, we selected the Labridae family (*n*=559 species) with a median age of 76±10 Myr obtained from the dated phylogeny. For the corals, we considered the Acroporidae family (*n*=274 species) also with a global distribution and with the earliest fossil in the data set dating to 131±5 Ma.

To compare simulated past biodiversity patterns to empirical data, we used fossil occurrences of corals (Supplementary Fig. 17) obtained from PaleoDB (www.paleodb.org). We mapped ancient coral species richness for three epochs with sufficient fossil occurrences, the Miocene (20 Ma), Eocene (40 Ma) and late Cretaceous (70 Ma). We retained only occurrences of fossils that were identified at the species level (that is, 25,077 species occurrences). Occurrences were converted into diversity maps by computing the sum of species present in the surrounding 40° × 40° window of each 5° × 5° cell when the fossil date range overlapped with the date of interest. Diversity maps were rescaled between 0 and 1.

### Landscape-based model of biodiversity dynamics

The use of correlative approaches in macroecological studies does not provide a mechanistic understanding of the way historical processes have shaped the distribution of alpha- and beta-diversity. To disentangle causalities for emergent patterns, the application of dynamic simulation models provide more understanding on the processes than statistical approaches^8^,^19^. In addition, phylogeography and diversification models are generally calibrated without information on the distribution of paleo-habitats and biogeographic history^45^,^46^. Ideally, diversification analyses should consider the historical sequence of habitat distribution, which implies to explicitly integrate landscape dynamics in the model. Accounting for shifting habitat through time within a dynamic model of speciation and dispersal was proposed by Gotelli *et al*.^8^ under the term general simulation model. General simulation model should track the presence (1) or absence (0) of a given species in a particular grid cell, the phylogenetic history of lineages, and historical biodiversity information which can be compared with palaeontological or phylogenetic evidences^8^. Nevertheless, this idea was so far never applied to real data, possibly because of the lack of habitat reconstruction for deep time periods^47^.

Here, we develop a diversification model constrained by shallow tropical reef maps. The simulation starts with a unique ancestral species and new species originate either within (that is, sympatric) or adjacent to (that is, parapatric) the geographical range of its ancestor. In early Cretaceous times (∼140 Ma), a continuous passive continental margin along the northern coast of Gondwana provided less opportunity for geographical separations with limited faunal beta-diversity^48^. We selected the simulation start that should be the most representative of all the possible lineages arising from the breakup of Gondwana. For younger clades, for example, those arising in the Miocene, it would be more appropriate to start in a subregion of an earlier time step and assess the sensitivity to the starting point. The model starts 140 Ma with one species occupying the entire available suitable cells along the coast of Gondwana which represented a well-connected habitat. Yet, other distant regions not connected to Gondwana likely harboured allopatric lineages that also contributed to extant biodiversity especially in the IAA (Supplementary Fig. 18).

The model records at any single point in time within a data frame the distribution of each species in each cell, together with its genealogy. During each million year time slice the simulation performs two phases:

The first phase is the speciation. We considered two modes of speciation, parapatric and sympatric implemented within two alternative models as well as a model combining both modes of speciation (the combined model hereafter). We refer to parapatric speciation because it is mainly dispersal limitation and not geographic isolation which drives divergence and speciation. Parapatric speciation arises when a species range is split into two or more distinct areas separated by a sea distance larger than *d*_s_ (Supplementary Figs 1 and 2). This mode of speciation was modelled as a cluster splits using cluster optimization algorithm based on the ‘dbscan' function in the fbc R library^49^. If the range of a species *i* is fragmented into *n* patches separated by a given distance threshold of *d*_s_, the species will separate into *n* species with smaller ranges (Supplementary Fig. 2). The model assumes that discontinuous range after 1 million years of separation generates full speciation with no possibility of hybridization or introgression. The main process generating species with the parapatric model is the successive phases of habitat patch disconnection and reconnection (Supplementary Fig. 2). For the sympatric speciation mode, sympatric speciation arises stochastically in a cell occupied by a species *i* with a probability *P*_s_ (Supplementary Fig. 3). Consistent with the speciation-area- hypothesis^12^, species occupying a large area, *A*, will show higher sympatric speciation frequency because *S=P*_s_ × *A* (Supplementary Fig. 19).

The second phase is the dispersal. The species in the time step *t* are allowed to disperse to all habitat cells at the time step *t*+1 that are distant by a threshold value lower than *d* from a Weibull dispersal kernel. This threshold is randomly sampled for each species at each time step. If a given species does not find a habitat cell within the dispersal distance, it will go extinct only if all the habitat cells inhabited by the species at time *t* disappear at time *t*+1.

The model can account for the parapatric mode (two parameters), the sympatric mode (two parameters) or both (three parameters) and can make inferences about assemblage properties (alpha-, beta-diversity), diversification rates (that is, speciation and extinction rates) and phylogenetic branching (that is, phylogeny). We run the simulation for a range of dispersal (*d*∈{1°:40°}) and speciation (*P*_s_∈{1e^−5^:1e^−4^}, *d*_s_∈{1°:40°}) rates. Values beyond those ranges were unrealistic as they predict no species (full extinction), too many species (for example, >20,000, especially when *d*>*d*_s_) or predict all species everywhere. The model was run until 1 Ma (and not to the current period) since we were interested in highlighting the geological contingencies on tropical reef assemblages. The effects of glaciations during the Quaternary (2.6 Ma to Present) have been studied elsewhere^3^. Therefore, we did not consider Quaternary climatic fluctuations in the current study. Parameter combinations (*d*, *d*_s_, *P*_s_) were run 20 times, the stochastic component in the dispersal kernel of the parapatric model and a larger stochastic component linked to cell speciation probability in the sympatric model. We ran a total of 16,000 simulations.

We highlighted the best model (BM) among all simulations across a set of empirical validations including (1) current alpha and beta-diversity of corals and fishes globally and for the Central Indo-Pacific region, (2) past species richness 70, 40 and 20 Ma based on fossil coral data (Fig. 2; Supplementary Figs 4 and 20). For fossil coral data, we focused on those three time periods because they were the most differentiated and contained the most available fossil information. We compared observed and predicted current beta diversity patterns using the pairwise Jaccard's dissimilarity index. We compared observed (for corals and fishes) and predicted species richness using a simple regression model from which we extracted the coefficient of determination (*R*^2^). Because models differed in the number of estimated parameters, we also computed the Bayesian information criterion (BIC) of each simulation as follow:


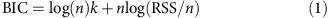


Where RSS represent the difference between scaled observed and predicted cell values, *n* the number of samples and *k* the number of model parameters. For each of the comparisons (fossil diversity gradients, current alpha and beta diversities), we summed the BIC to rank the simulations (Supplementary Fig. 21). We obtained general models of marine faunal dynamics explaining shared properties in the historical biodiversity dynamics of both corals and fishes.

### Nestedness and species turnover patterns

The IAA also called the coral triangle (that is, the Indonesian archipelago, the Malaysian peninsula, the Philippines, New Guinea and northern Australia) is widely recognized as the global centre of modern marine biodiversity^50^. At the scale of the Central Indo-Pacific region, the number of reef fishes and corals was found to decrease with increasing distance from the coral triangle, with the tendency that distant assemblages being nested within those of the coral triangle^19^,^21^,^51^. We therefore evaluated if the parapatric and sympatric models faithfully reproduced this biogeographical pattern. To do so, we used the NODF index based on paired overlap and decreasing fill^52^ to measure the extent to which species present in species-poor cells constitute proper subsets of those ones present at species-rich cells. More specifically, we calculated the NODF index among sites (hereafter NODF_sites_) that ranges from 0 (cells are not nested) to 100 (cells are perfectly nested). To determine the probability that nestedness could be obtained by chance, we compared the observed NODF_sites_ value to those obtained by a null model where species-presence matrices are randomly drawn (*n*=9,999) under the sequential swap algorithm^53^. Last, we extracted the rank order of sites in the maximally packed matrix (that is, maximally ordered) for simulated occurrence data under the parapatric and sympatric models and observed fish occurrence data. We assessed the level of congruency between observed and predicted rank orders using a Spearman's rank correlation test.

Because differences in species composition between places may result from both nestedness and species turnover (that is, the replacement of some species by others between assemblages), we also quantified the levels of species turnover across the Central Indo-Pacific region using a pairwise turnover metric (*β*_jtu_) that is independent of species richness differences^54^. We quantified the level of congruency between observed and predicted distance matrices using a Mantel test (999 permutations). To visualize observed and predicted patterns of species turnover, we plotted the corresponding distance matrices along reduced axes using the non-metric multidimensional scaling neighbour-joining algorithm. The ordination results were then plotted and mapped by assigning a colour to each grid cell according to its position in the ordination space.

### Phylogenetic reconstruction from molecular sequences

We reconstructed the phylogenetic relationships of species occurring in tropical reefs of the Labridae family together with outgroups according to previous phylogenies^55^. Molecular sequences from published sources were downloaded on GenBank focusing on the most frequent loci (Supplementary Data 1). Those loci were then concatenated to perform a supermatrix approach for phylogenetic reconstruction with sufficient taxon overlap for each loci. We used four mitochondrial gene regions (non-coding: 12S and 16S, coding: Cytb and CO1) and three nuclear genes (RAG2, S7 II and tmo4c4), getting data for 55% of known species. Each marker data set was independently aligned using the algorithm MUSCLE with standard settings and alignments were then manually edited using MEGA 6 (ref. 56). We tested substitution models using a maximum likelihood approach with a random starting tree for each gene alignment and we selected the best models using the BIC criterion on Jmodeltest 2.1.6 (refs 57, 58; Supplementary Table 1). Alignments were afterwards concatenated in a supermatrix using SequenceMatrix^59^. We then analysed the supermatrix using a Maximum Likelihood approach via the algorithm RAxML (ref. 60) implemented in RAxMLGUI (ref. 61). We performed 10 parallel runs to fully explore tree space and to avoid being retained on a local optimum. We then selected the best tree using likelihood scores. To estimate node ages, we used the penalized likelihood method on the best likelihood tree. We then used the ultrametric tree as a starting tree for Bayesian inference of tree topologies and node ages using Markov chain Monte Carlo (MCMC) in BEAST v1.8.1 (ref. 62). We ran six independent MCMC, 40 × 10^6^ steps long under individual gene models previously selected for each reconstruction. We used the same fossil and biogeography based calibrations of Cowman and Bellwood^63^ as we had no update in fossil calibrations since then, and there is no fossil data available to our knowledge for the additional families (Supplementary Table 2). We checked for the convergence and stationarity of each independent run using Tracer v1.6 (ref. 64). We combined the six independent runs for each data set (after removing an appropriate burn-in) using LogCombiner v1.8.1 (ref. 62) to reach an effective sample size above 200 for all our estimates and we extracted the maximum clade credibility tree for combined tree sets using TreeAnnotator v1.8.1 (ref. 62). The resulting time-calibrated phylogeny (Supplementary Fig. 14) is consistent with previously published phylogenies for the Labridae, with a few differences. Overall, the node age estimates appear to be 5–10 million years older than previously estimated^40^ (Supplementary Fig. 22). In addition, few nodes showed low posterior probabilities (Supplementary Fig. 23).

### Biogeographic reconstructions using the phylogeny

Due to the lack of knowledge of the sister family of the Labridae, we confined our biogeographic analyses to within the Labridae. Based on a global-scale distribution database^3^,^44^, we classified the distribution of the species belonging to the Labridae into three main areas, namely Atlantic, Western Indian Ocean and Central Indo-Pacific. We performed ancestral biogeographic estimation on the reconstructed empirical phylogeny using the R package BioGeoBEARS^65^. BioGeoBEARS is designed to perform inference of biogeographic history on time-calibrated phylogenies using models of dispersal, extinction and cladogenesis (DEC)^66^ with the addition of several other biogeographic parameters that can be combined to form other biogeographic models (for example, DIVA, BayAREA) in a likelihood framework. In addition, it allows the inclusion of founder-event speciation scenarios through the inclusion of the ‘Jump' (+J) parameter. Alternate models can be compared using Akaike information criterion (AIC). We estimated the biogeographic range evolution under a DEC model which allowed many alternative biogeographic scenarios. In addition we compared the inclusion of the ‘+J' parameter for the empirical data using AIC. Species ranges were categorized into three separate biogeographic regions – Atlantic, West Indian Ocean and Central Indo-Pacific. To reflect the tectonic history of marine habitat we allowed a fourth area to only exist in the past – the Tethys. Lineages were only permitted to occupy, or include the Tethys region as part of its range before 12 Ma, a time that signifies the final closure of the Tethys seaway^67^. To reflect the fossil and paleogeographic record, we constrain the deeper nodes in the tree (before 70 Ma) to include the Tethys region in the node range estimation (but not exclude other adjacent area combinations). Dispersal was only allowed between adjacent areas and a dispersal matrix was constructed to reflect the formation of land bridges at different time period (closure of the Tethys Seaway – 12 Ma; Isthmus of Panama – 3.1 Ma). Recent geologic evidence points to earlier closures of the Isthmus of Panama^68^ and its extended evolutionary influence on marine and terrestrial biotas^69^. Here, we model our dispersal matrix on the final closure of the Isthmus, but earlier vicariance events are reported in the Labridae^70^. Under the DEC+J model, we identified the age of colonization of the central Indo-Pacific among the different lineages of Labridae.

### Diversification rates

To compare to the simulated diversification rate to empirical patterns, we estimated extinction and speciation rates shaping diversification for the coral family Acroporidae using fossils occurrences following the method (PyRate) proposed by Silvestro *et al*.^71^ This method includes the probability of preservation and sampling of fossils to estimate the lifespan of each lineage. We first removed all undetermined taxa from the data set. Due to the lack of fossil occurrences before 60 Ma, we only estimated diversification rates during the last 60 Myr. We first ran 10,000,000 BDMCMC generations both assuming constant and Gamma distributed preservation rate to test the heterogeneity of preservation rate. We then generated 10 randomization of the age of fossils occurrences and performed 10 independent BDMCMC analyses to estimate the diversification rates through time. We examined the posterior samples using Tracer v1.6 (ref. 64) and combined it after removing an appropriate burn-in to get a good estimation of speciation and extinction rates.

Because missing extinct lineages in the most ancient section of the phylogeny of Labridae preclude any diversification estimate in the Cretaceous from phylogenies, we computed the rate of diversification from a phylogeny containing 18 tropical reef fish families (that is, *Labridae* including *Scaridae*, *Pomacentridae*, *Chaetodontidae*, *Acanthuridae*, *Haemulidae*, *Balistidae*, *Carangidae*, *Serranidae*, *Lutjanidae*, *Sparidae*, *Caesionidae*, *Holocentridae*, *Mullidae*, *Muraenidae*, *Tetraodontidae*, *Lethrinidae* and *Siganidae*). We obtained this phylogeny by pruning a time-calibrated phylogeny for 7,822 extant fish species^55^. These families were selected as the most representative reef fish families, that is, they are abundant and speciose on tropical reefs^72^. These families are well sampled in the phylogeny of Rabosky *et al*.^55^ with more than 80% of known genus. As missing species may influence the estimation of diversification rates, we grafted those species on the pruned phylogenetic tree (1,291 missing species out of 2,224) based on published phylogenies for these families, supplemented by taxonomic information from fish identification guides and FishBase (www.fishbase.org). Specifically, new tips representing unsampled species were added to direct sister species when information allowed or to the base of the clade representing its genus. Using this empirical phylogeny, we estimated diversification rates based on an evolutionary model under a birth-death-shift process^46^. The birth-death-shift model uses a likelihood approach to estimate speciation and extinction rates like in a simple birth-death process but it also allows changes of those parameters through time if we force diversification shifts to happen at given times. For a given number of shifts, we simultaneously estimated time at which shifts occur, and speciation and extinction rates within each time interval between diversification shifts. We calculated those parameters for several numbers of shifts and we compared the resulting models using the AIC. The method proposed by Stadler^46^ is robust to phylogenetic uncertainty due to polytomies. Finally, we extracted the simulated values of diversification rate expected under the parapatric and sympatric models by computing for each time step the speciation rate minus the extinction rate and we compared the observed diversification rates with those obtained from the observed phylogeny for fishes and fossils for corals (Supplementary Fig. 8).

### Data availability

The R script to run the species diversification model together with the bathymetry reconstructions are available online on FigShare (DOI: 10.6084/m9.figshare.3081226). Species diversity data and fossils are made available through the IUCN http://www.iucnredlist.org/ and http://fossilworks.org/.

## Additional information

**How to cite this article:** Leprieur, F. *et al*. Plate tectonics drive tropical reef biodiversity dynamics. *Nat. Commun.* 7:11461 doi: 10.1038/ncomms11461 (2016).

## Supplementary Material

Supplementary Information Supplementary Figures 1-23, Supplementary Tables 1-2 and Supplementary References

Supplementary Data 1 Genbank accession numbers for the sequences used in the phylogenetic reconstruction. Species in grey are the species for which we found no suitable sequence to use.
