# Supplementary material for: Plate tectonics drive tropical reef biodiversity dynamics
Source: Nat Commun. 2016 May 6;7:11461. doi: 10.1038/ncomms11461 (PMC4859061; doi:10.1038/ncomms11461)
Supplement: Supplementary Information — Supplementary Figures 1-23, Supplementary Tables 1-2 and Supplementary References [file ncomms11461-s1.pdf]

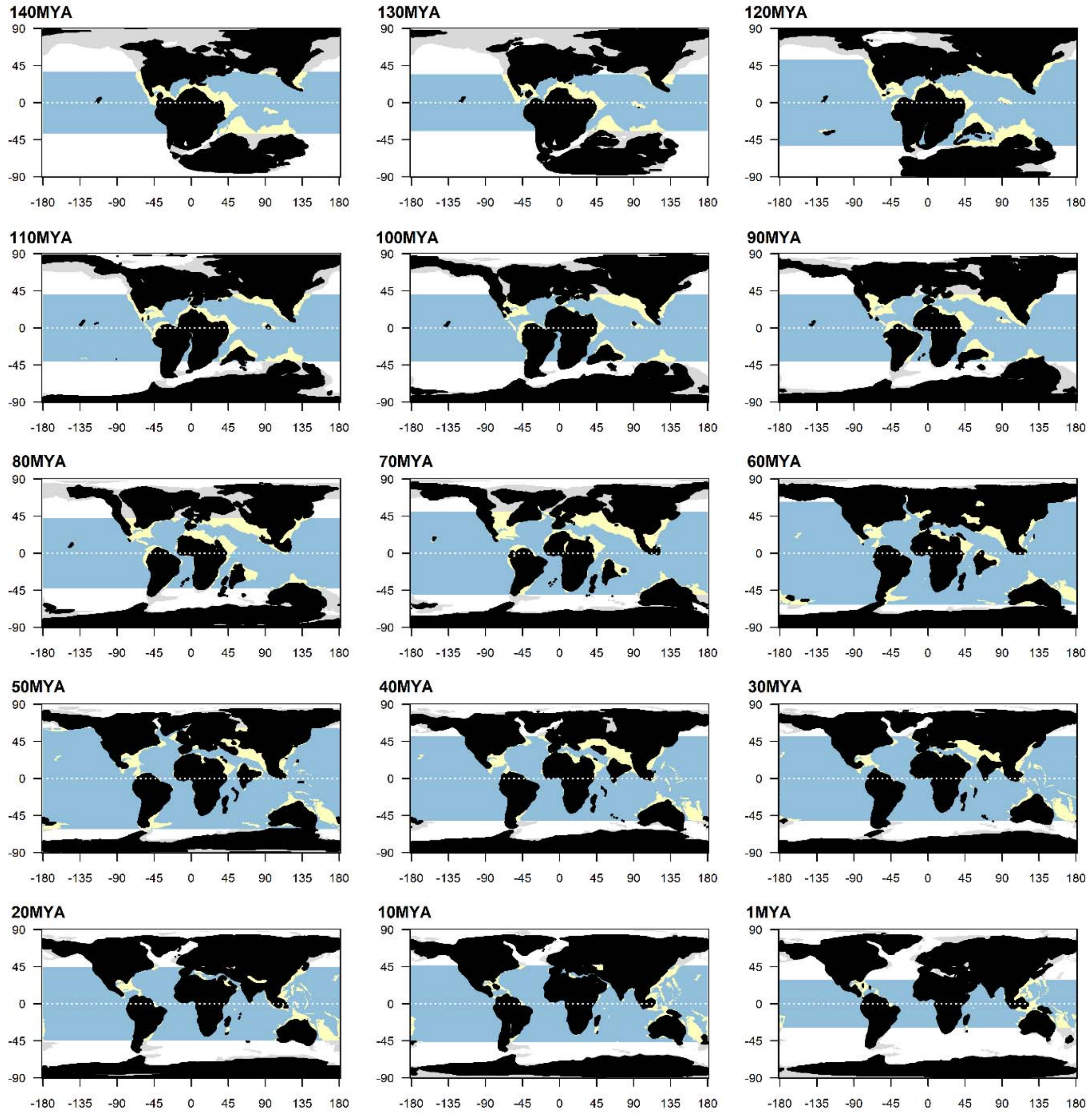

Supplementary Figure 1: Distribution of shallow and deep ocean sea floor across the past 140 Ma. The latitudinal tropical limit was obtained from the fossil distribution of coral species. Light blue represents deep tropical ocean, while yellow represents shallow tropical reefs at a temporal resolution of 1 Ma and spatial resolution of 1° (only 10 Ma time steps are shown here). White and light grey represent deep ocean and shallow waters outside the tropical boundary, respectively.

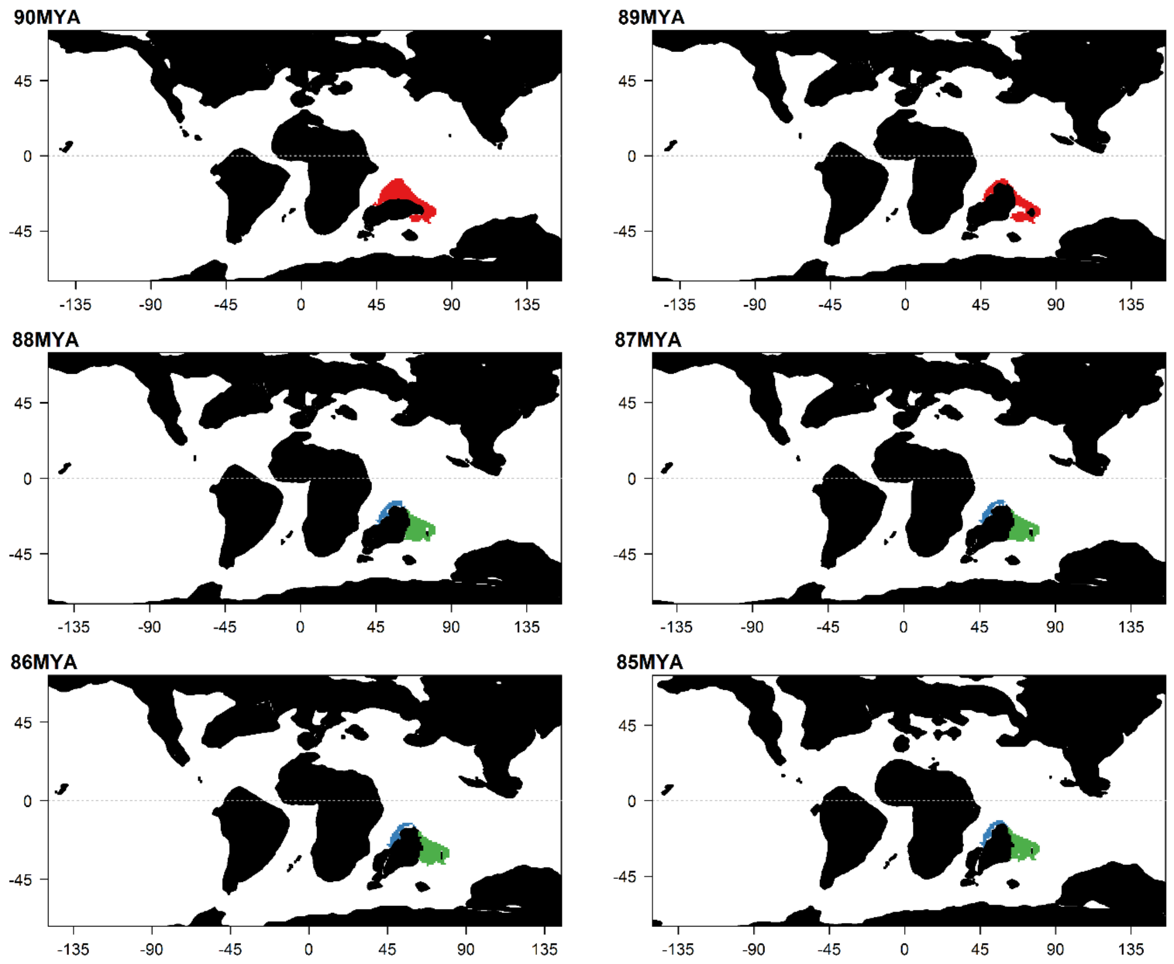

Supplementary Figure 2: Illustration of the functioning of the parapatric speciation model. Shown is a hypothetical speciation event occurring around Paleo-India through parapatric speciation across a series of time steps. When the range of the red species becomes disconnected into a south-eastern and a north-western cluster and if the distance between these two parts is larger than the distance threshold d_s, those clusters will become new separate species, the “blue” and “green” species. If those species encounter each other in the same area later, they will be treated as two entirely different species and form a species assemblage.

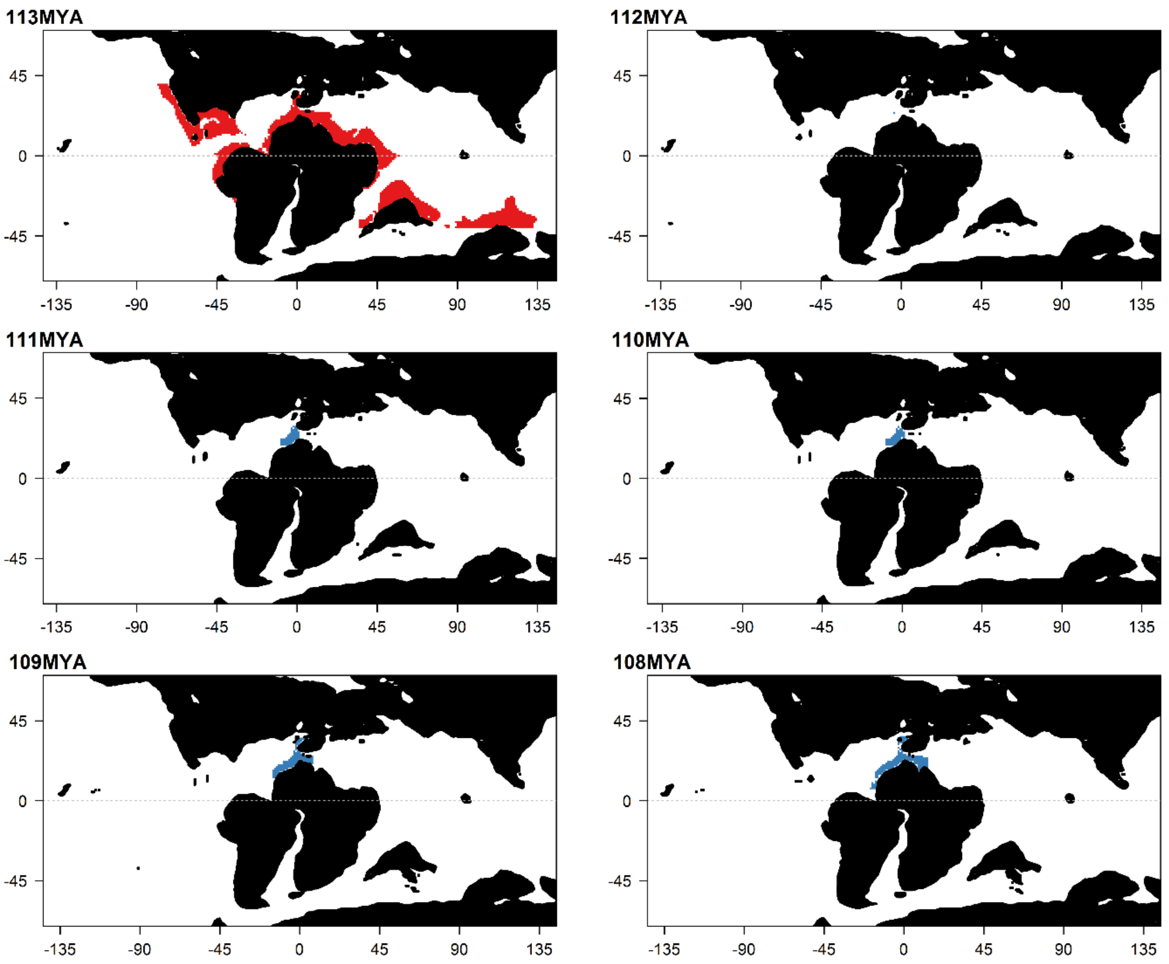

Supplementary Figure 3: Illustration of the functioning of the sympatric speciation model. Shown is a hypothetical sympatric speciation event occurring in the Tethys above paleo-Africa. From the red species with a widespread distribution range, a sympatric speciation event happens with probability equal to p_s in one cell between paleo-Europe and paleo-Africa and gives birth to the new species "blue". Then this species colonizes available habitats according to the dispersal rate *d*.

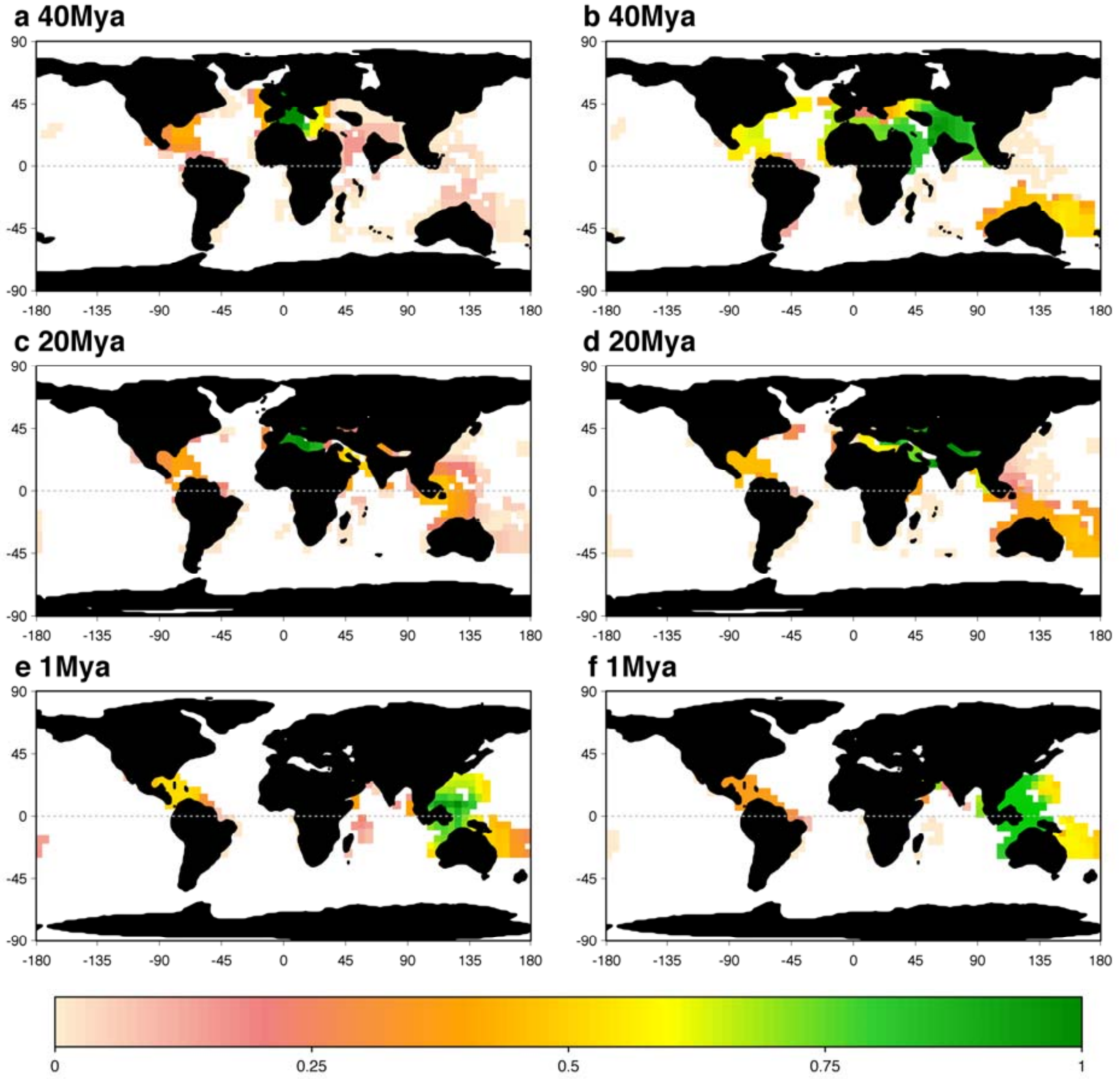

Supplementary Figure 4 Hopping biodiversity hotspots. Shown are the results observed for coral fossil diversity (a, c, e) and of the simulation with the sympatric model (b, d, f) for three time periods Eocene (a, b) Miocene (c, d), Quaternary (e, f). The two most ancient time periods depict observed diversity from coral fossil records (www.paleodb.org), while the most recent period shows contemporary coral diversity pattern (IUCN). The best model provided good correlation with Eocene and Miocene biodiversity patterns (d =5, p_s=6e-5, 40Ma : R^2=0.12; 20Ma : R^2=0.13) and with contemporary biodiversity (d =5, p_s=6e-5, fish: R^2=0.46; coral: R^2=0.44).

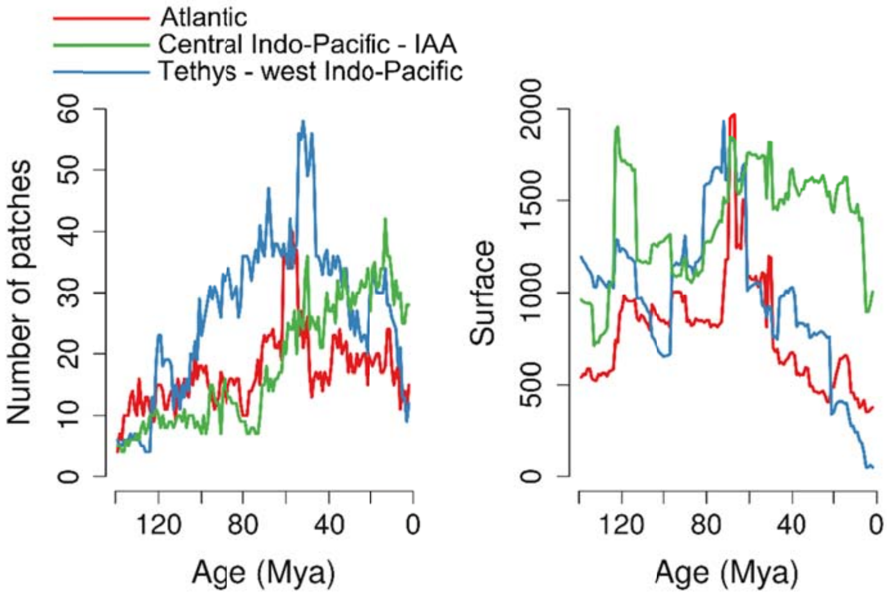

Supplementary Figure 5: Number of patches and total surfaces through time. Shown are trends for the Atlantic (red), Tethys, West Pacific (blue) and Central Indo-Pacific (CIP) including the Indo-Australian Archipelago (IAA) (green). In the Cretaceous and Eocene, the Tethys Ocean contained larger shallow reef surfaces, which was patchily distributed. The Central Indo-Pacific shows a steady increase in the number of patches with a recent peak around 15 Ma.

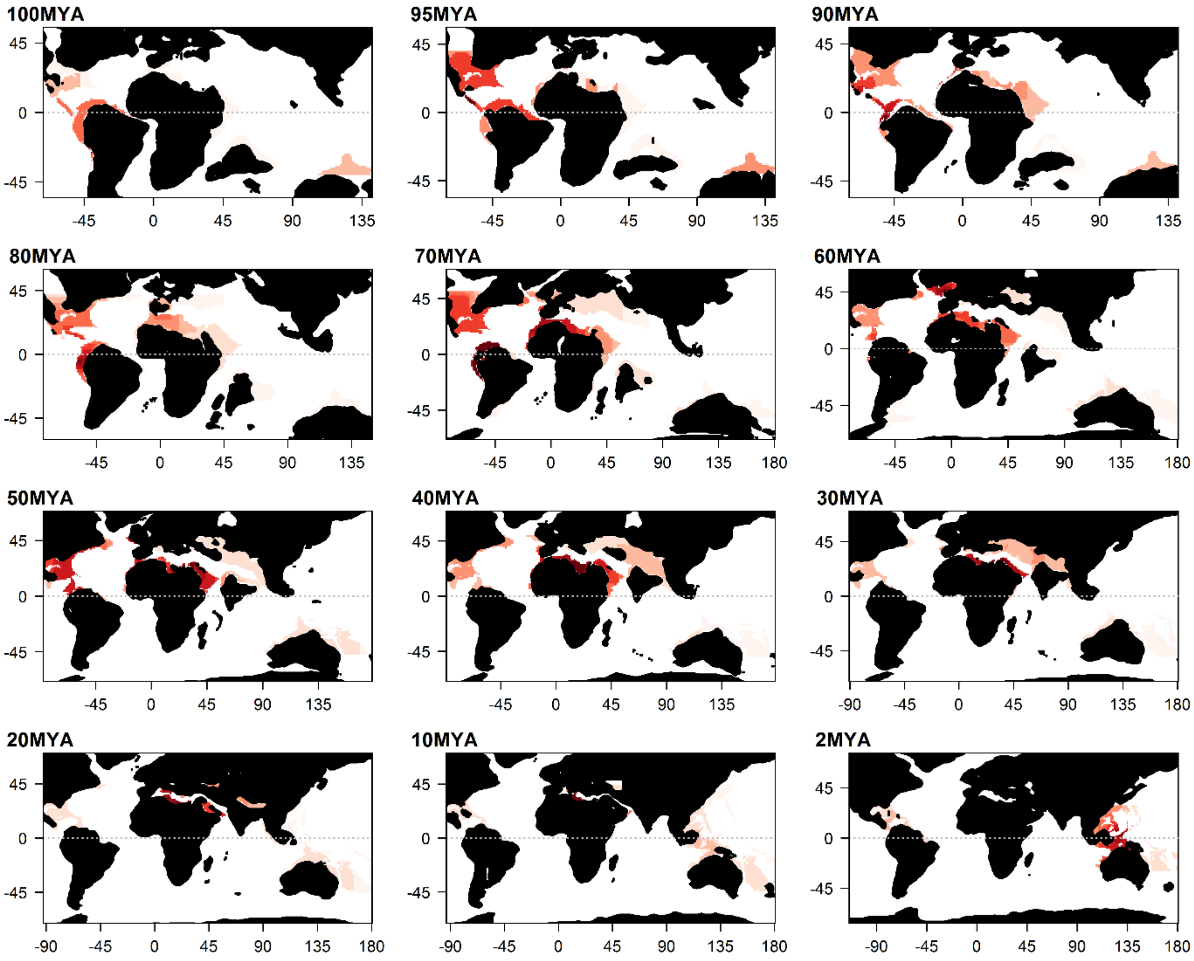

Supplementary Figure 6: Simulated species richness through time. Results are based on the best model of parapatric speciation using parameters d=4 and d_s=5 across the last 100 Ma. The colour gradient from light red to dark red represents the richness gradient from low to high rescaled between 0 and 1 for each time step. A lineage in the central Tethys had a high diversification especially between paleo-Europe and paleo-Africa. A small portion of the lineage migrated to the central Pacific, while some lineages in the Mediterranean region got extinct at the closure of the Mediterranean Sea. One Australian lineage separated early after the northward movement of the subcontinent of India. It should be noted that each figures has an independent colour gradient, and the shade might vary depending on the difference between the richest and poorest cell at a particular time step. In particular, the increase in the IAA at 2 Ma occurs because of the strong extinction in the Mediterranean Sea after the temperature cooling, which made the IAA the new global hotspot.

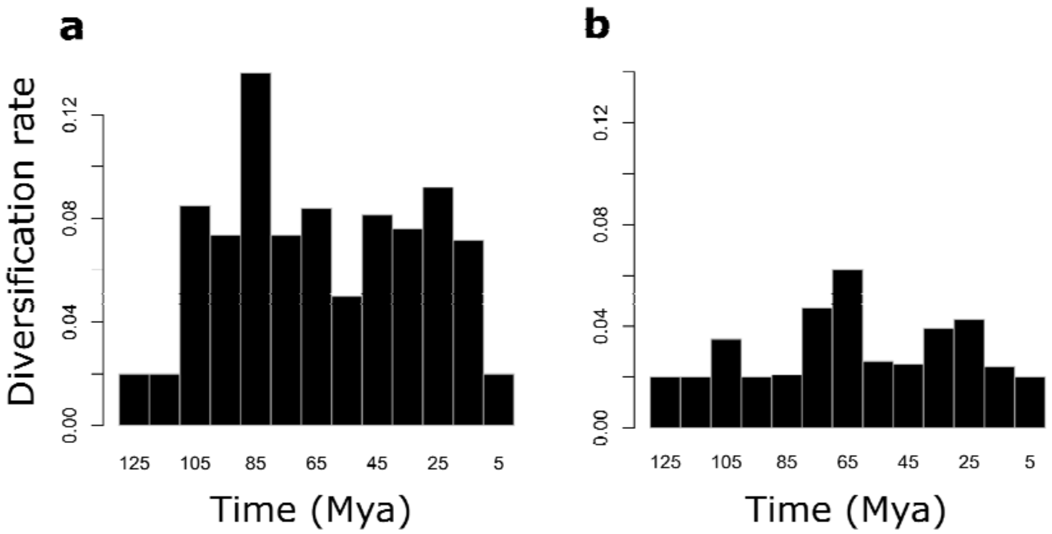

Supplementary Figure 7: Simulated diversification rates. Shown are a) Simulated diversification rate through time inferred from the parapatric model as speciation rate minus extinction rate. The parapatric speciation model (d=4, d_s=5) predicts a peak of diversification around 85 Ma, corresponding to the high degree of fragmentation in the Tethys sea, b) while the sympatric model (d=5 and p_s=6e-4) predicts a diversification peak later around 65 Ma.

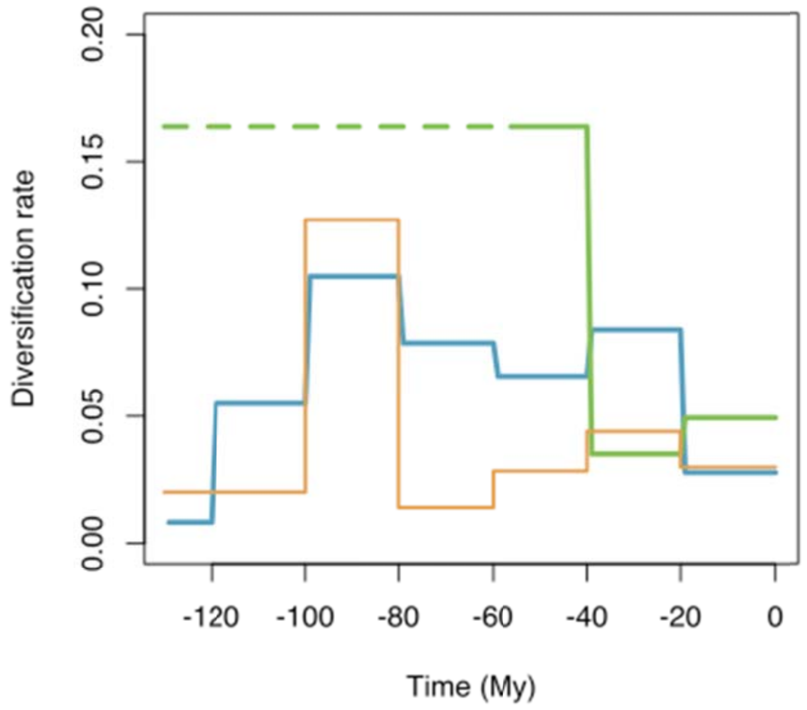

Supplementary Figure 8: Simulated and observed diversification through time. Simulated diversification rate (in blue) compared to the diversification rate estimated from the fish phylogeny (in orange) and from the coral fossils (in green). Because of poorer fossil records of Acroporidae earlier than 60 Ma, the uncertainty is higher as indicated by the dashed line. The full methods are presented in the supplementary methods. The empirical patterns found for fish and coral agree with a higher diversification rate before the Eocene as shown in the simulation with the parapatric model (*d*=4 and *ds*=5).

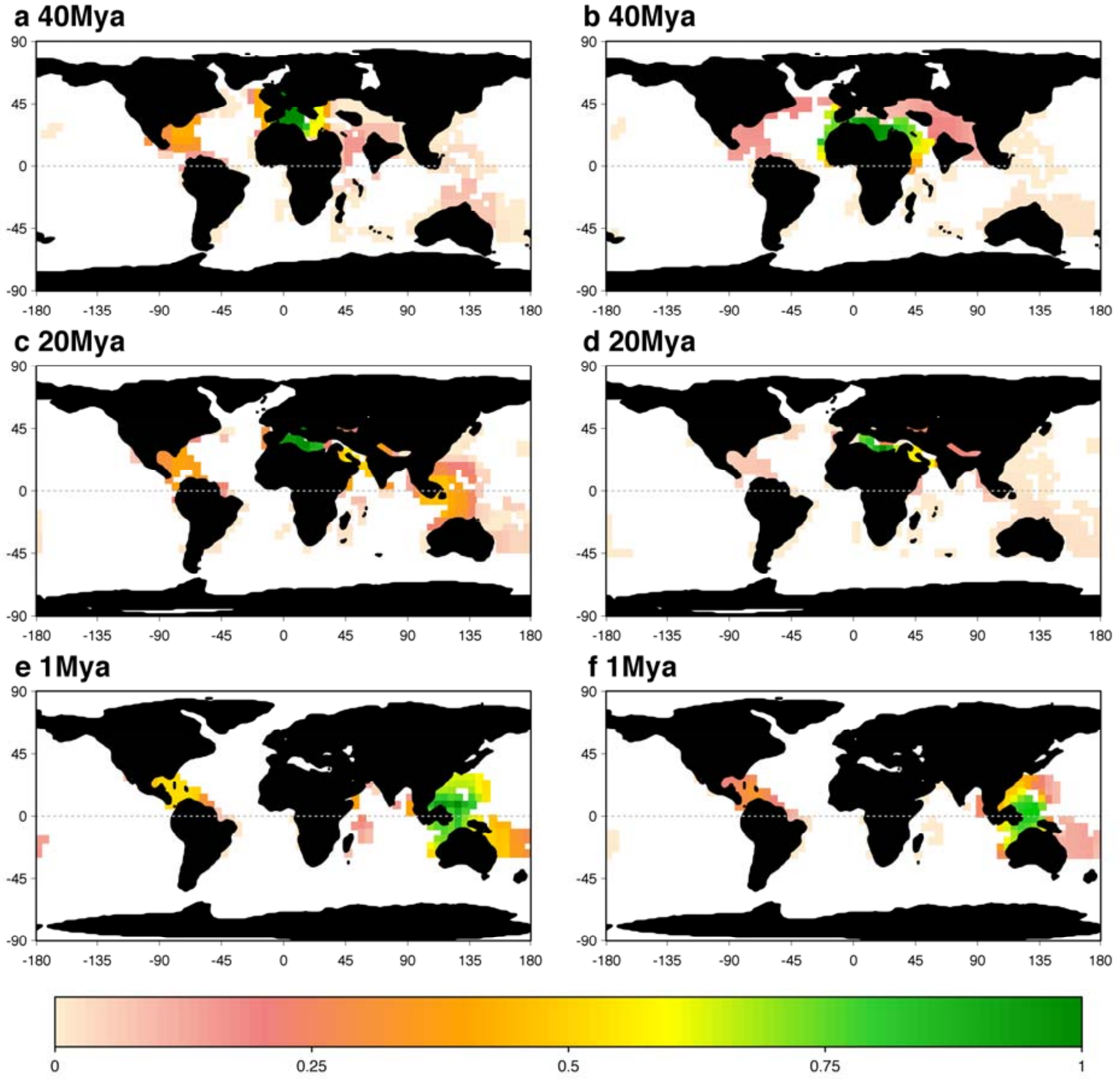

Supplementary Figure 9: Hopping biodiversity hotspots. Shown are the results observed from coral fossils (a, c, e) and of the simulation with the parapatric model with *d*=4 and *ds*=5 (b, d, f) for three time periods Eocene (a, b), Miocene (c, d), and Quaternary (e, f). The two most ancient time periods depict observed richness interpolated from coral fossil records (www.paleodb.org), while the most recent period shows contemporary coral richness (IUCN). Richness values were rescaled between 0 (minimum, pink) and 1 (maximum, green). The parapatric model was forced with high a degree of extinction 66 Ma (80% of extinct species). Hopping hotspots simulated with the parapatric model show the same pattern as without extinction indicating the robustness of the pattern to a mass extinction event.

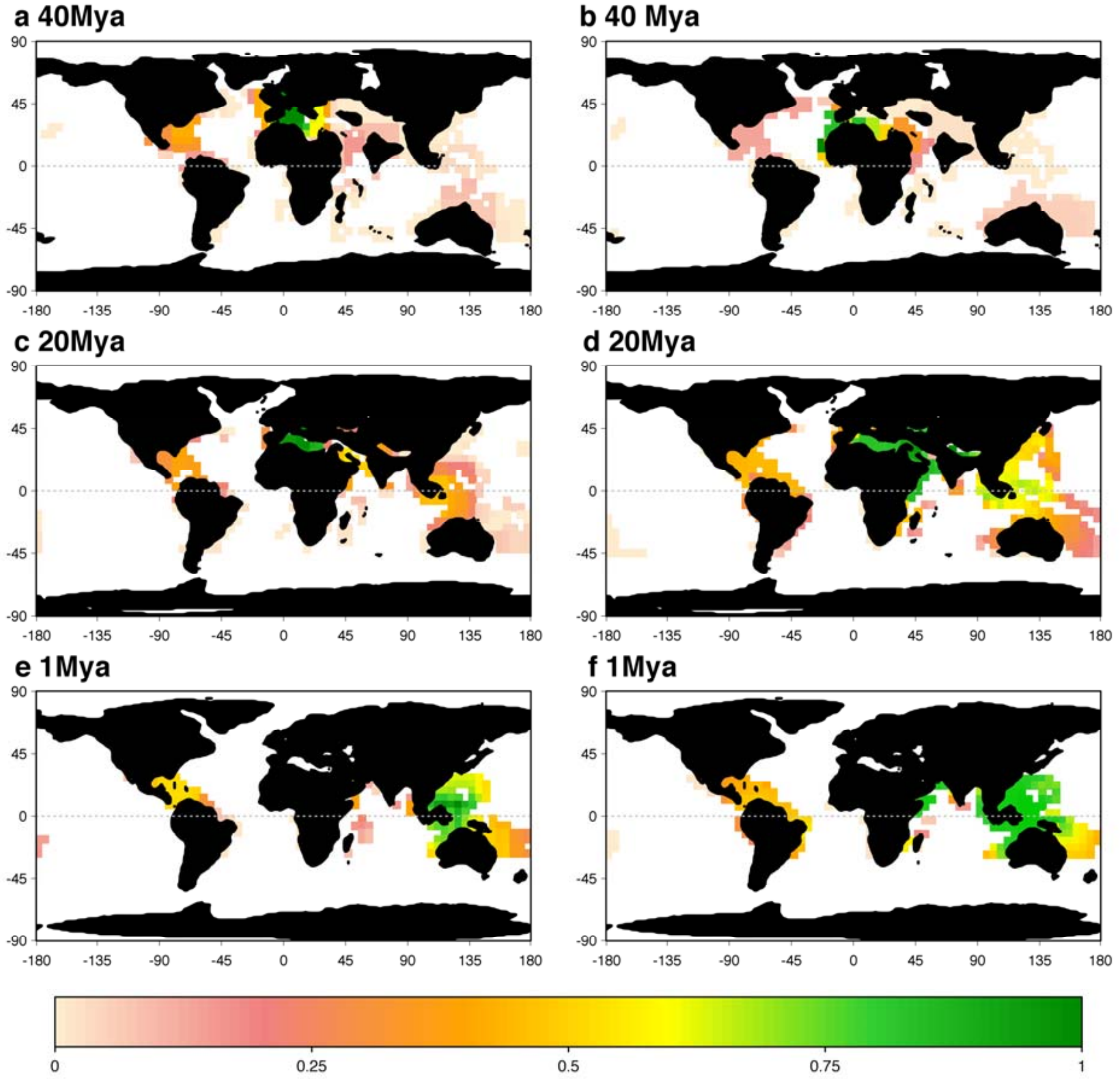

Supplementary Figure 10: Hopping biodiversity hotspots. Shown are the results observed from coral fossils (a, c, e) and simulated with the parapatric model with *d*=4 and *ds*=6 (b, d, f) for three time periods Eocene (a, b), Miocene (c, d), Quaternary (e, f). This simulation used the second plate kinetic model (model 2 see methods) initiating the simulation 140 Mia accounting for True Polar Wander from 140 to 100 Ma. Here we show results from the Eocene since biological information for model validation are scarce prior to this period. The two most ancient time periods depict observed richness interpolated from coral fossil records (www.paleodb.org), while the most recent period shows contemporary coral richness (IUCN). Richness values were rescaled between 0 (minimum, pink) and 1 (maximum, green). The best model provided good correlation with Eocene and Miocene (d=4, d_s=6,

40 Ma: R^2=0.19; 20 Ma: R^2=0.37) and with present-day diversity (*d*=4, *ds*=6, fish: R^2=0.27; coral: R^2=0.29).

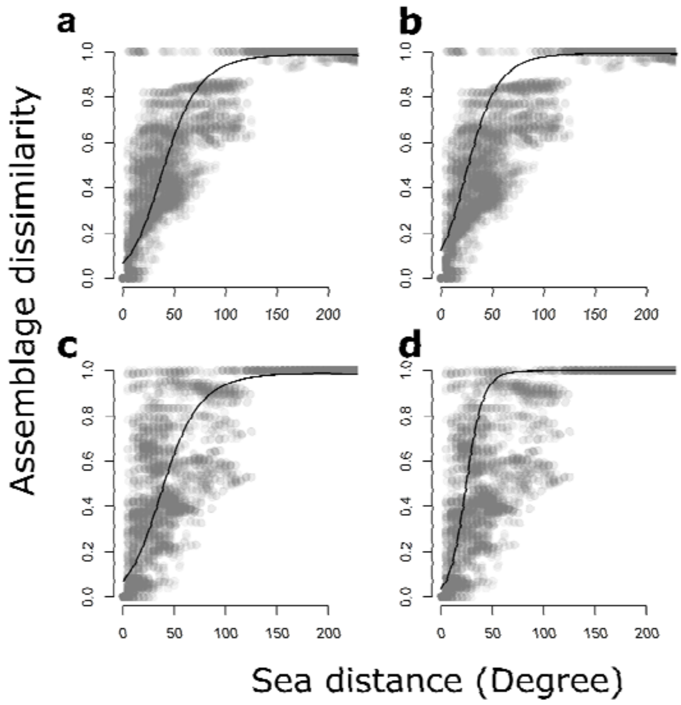

Supplementary Figure 11: Observed and predicted patterns of beta diversity. The X axis represents a gradient of sea distance accounting for the shape of land masses. The points represent observed values of assemblage dissimilarity for fishes of the Labridae family (a, b) and corals of the Acroporidae family (c, d). The black line represents the fitted relationships between assemblage dissimilarity and geographical distance for the sympatric (a, c) and parapatric (b, d) models. As distance increases between cells, fish assemblages are less similar from each other. Observed and predicted values of assemblage dissimilarity were strongly correlated globally when considering both the parapatric (d=4, d_s=5, fish: r_m=0.63; coral: r_m =0.56) and sympatric (d =5, p_s=6e-4, fish: r_m =0.77; coral: r_m =0.76, Extended Data Fig. 6) models. Observed and predicted values of assemblage dissimilarity were also correlated when only considering the Central Indo-Pacific region (parapatric: d=4, d_s=5, fish: r_m =0.22, coral: r_m =0.29, sympatric: d=5, p_s=6e-4, fish: r_m =0.12, coral: r_m =0.26).

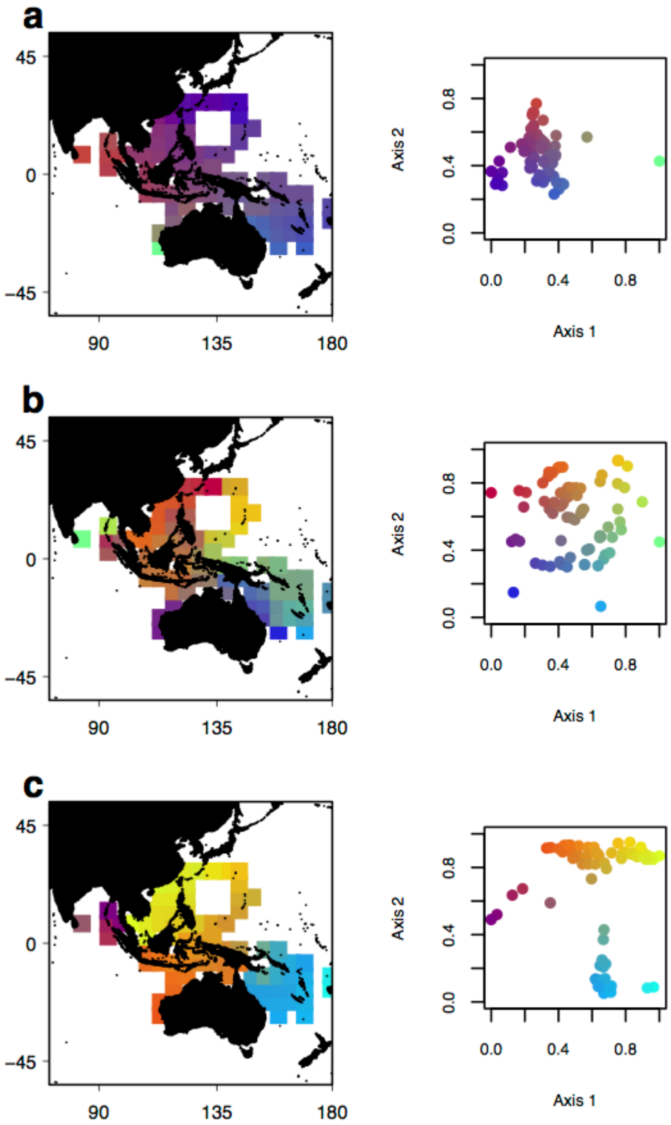

Supplementary Figure 12: Observed patterns of species turnover in the Indo-Australian Archipelago. Shown are results for fishes (a) and corals (b) and those predicted by the parapatric model with *d*=4 and *ds*=5 (c) based on a NMDS ordination diagram for the Central Indo-Pacific region. The colour gradients represents the position of the points within the ordination space. Both observed and predicted patterns revealed a marked species turnover from west to east (Malaysia to the east of Australia), but also north to west (Japan area to the coasts of Australia). A Mantel test shows a

significant correlation between observed and predicted values of species turnover for both corals (r_m=0.33, P<0.001) and fishes (r_m=0.44, P<0.001).

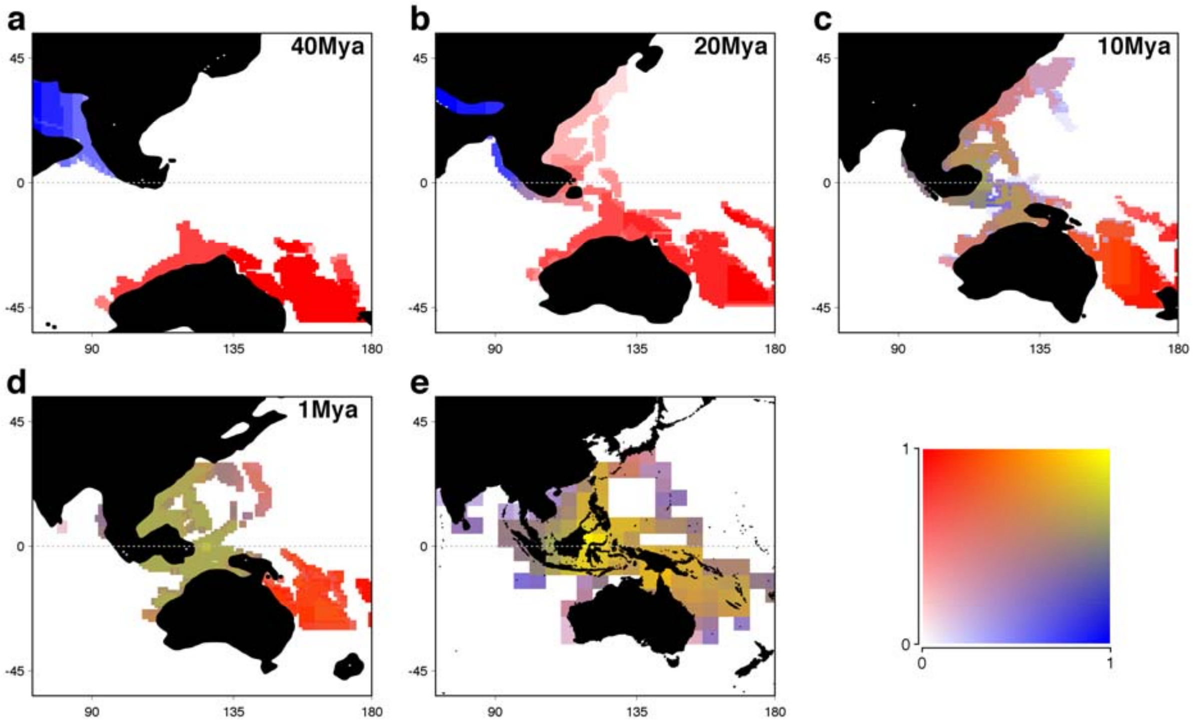

Supplementary Figure 13: Biodiversity dynamic through time. Shown are biodiversity through time (a,b,c,d) predicted by the sympatric model (d=5, p_s=6e-4) for the Tethyan (blue) and Australian (red) lineages in the Central Indo-Pacific region. The merging of these two lineages is represented by the yellow colour. e) Observed contemporary richness of the Tethyan (scarinae in blue) and Australian (hypsigenyines and pseudocheilines in red) lineages inferred from a biogeographic reconstruction (Supplementary Fig. 14).

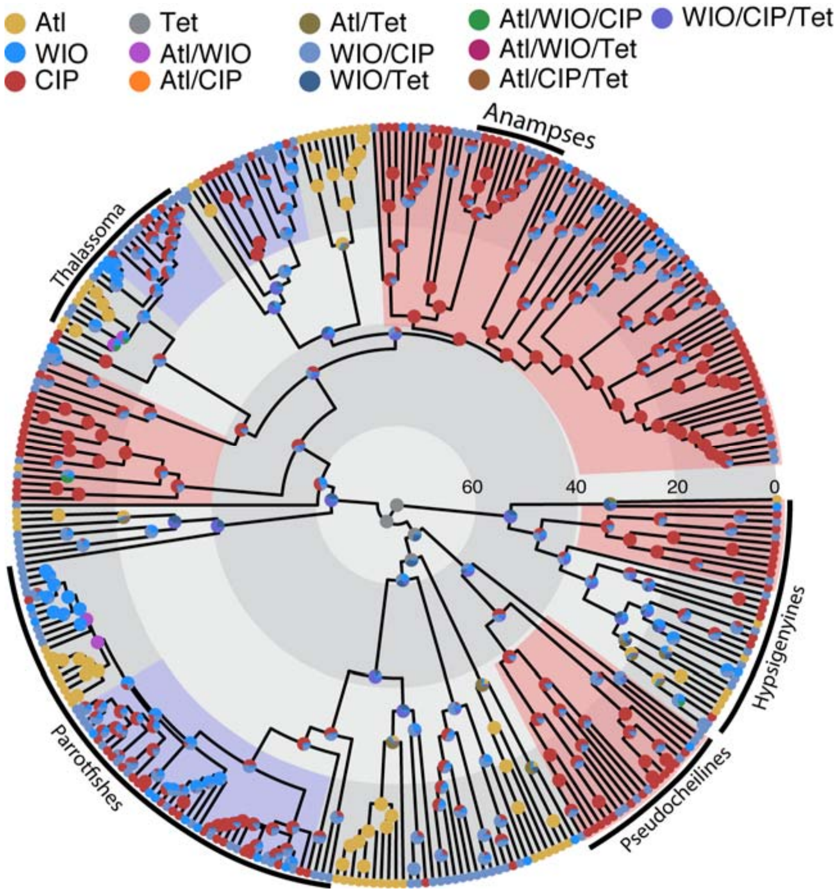

Supplementary Figure 14: Biogeographic reconstruction for the Labridae family. The labels represent the different regions (Atlantic: Atl, West Indian Ocean: WIO, Central Indo-Pacific: CIP) based on a DEC model (see Methods for more details). Lineages highlighted in red represent separation of possible lineage along the Australian coast earlier than the Miocene as suggested by the geographic reconstruction while lineages in blue indicate a more recent colonization of central Indo-Pacific (CIP 20 Ma). Within the Labridae, lineages of hypsigenyines and pseudocheilines became isolated in the Central Indo-Pacific region approximately 40 Ma (reconstructed state dominated by CIP >50% early in those lineages) and may have colonized the Australian coast through a paleo-islands network across the Southern Indian Ocean (Supplementary Figure 15). In contrast, the scarinae (parrotfishes) lineage with earliest fossils in Tethys likely represents a more recent colonization in the Miocene along the Indian coast (Supplementary Fig. 15).

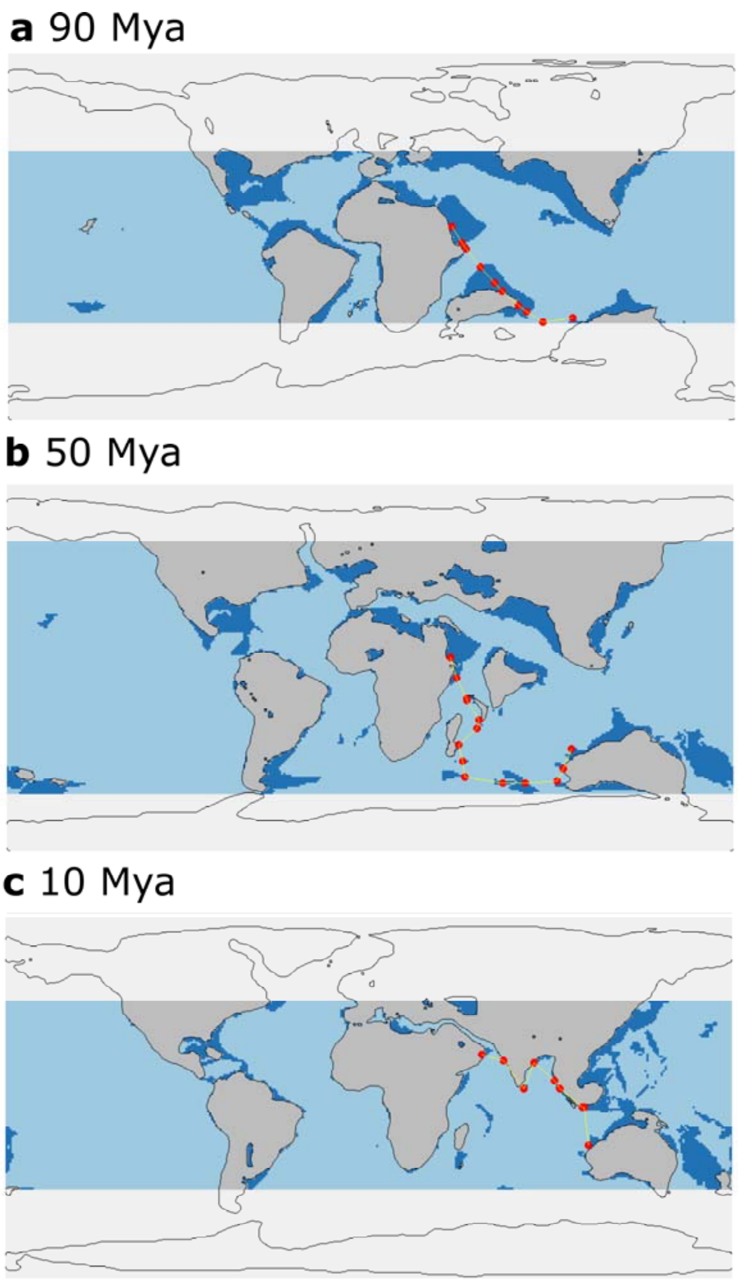

Supplementary Figure 15: Connectivity between the central Tethys and the Australian coast. Stepping stone optimisation analysis applied on the connectivity between central Tethys and Australia at three different time periods. Deep blue colour represents shallow reef habitat, while light blue deeper sea. We computed the path that allowed crossing from Eastern Africa to Australia via the shortest distance jumps indicated by the red dots. This model is based on the assumption that species may cross some long distance but only via intermediate stepping stones to send enough propagules onward. Shown are a) 90 Ma the optimal stepping stone path was around northern Greater India. b) More recently, 50 Ma, the optimal stepping stone path was through a southern Archipelago near

Madagascar, the Reunion and Kerguelen volcanic hot spots. Those first two pathways are supported by the presence of paleo-endemics near Madagascar. c) 10 Ma the optimal stepping stone path was along the coast of India. This corresponds to three possible waves of colonisation of the Central Indo-Pacific.

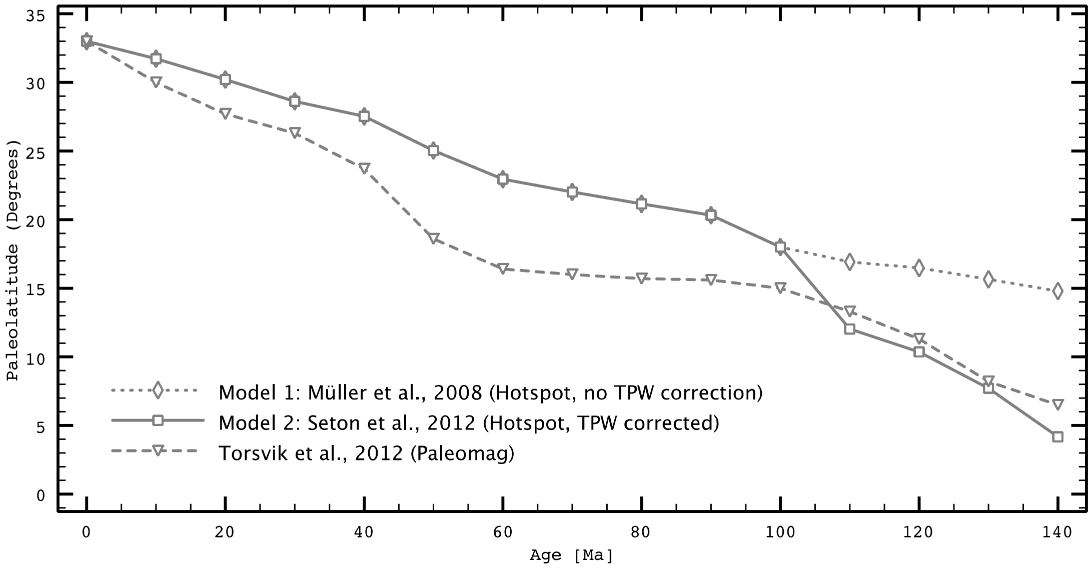

Supplementary Figure 16: Difference in paleolatitudinal position among different reference frames. This plot shows the difference in paleolatitudinal position for a point on the northern edge of the African plate at 20˚E 33˚N predicted by absolute moving hotspot reference frames[1], a hybrid absolute moving hotspot reference frame corrected for TPW for the times older than 100 Ma[2], and a pure paleomagnetic reference frame[3]. The largest difference between the predicted positions is about 800 km at around 60 Ma.

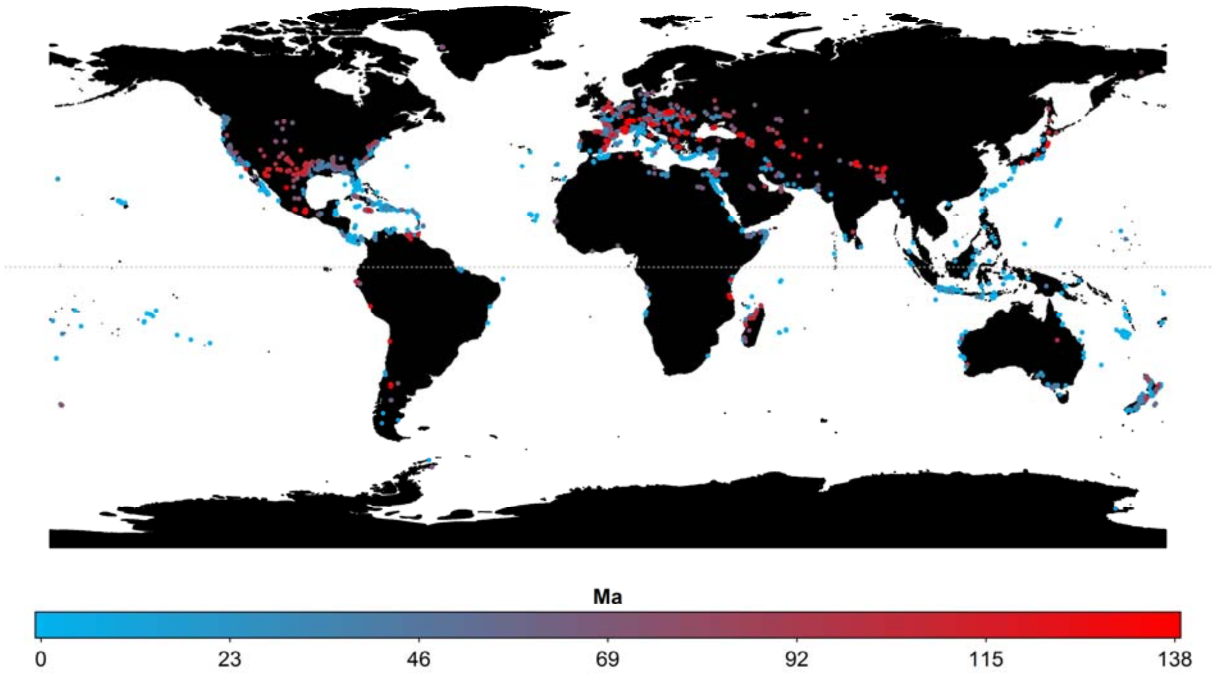

Supplementary Figure 17: Plot of scleractinia fossil occurrence. Fossil data were obtained from www.paleodb.org and used to estimate the tropical borders and the coral fossil diversity. The colours from blue to red indicate a gradient from the most recent fossils to the most ancient.

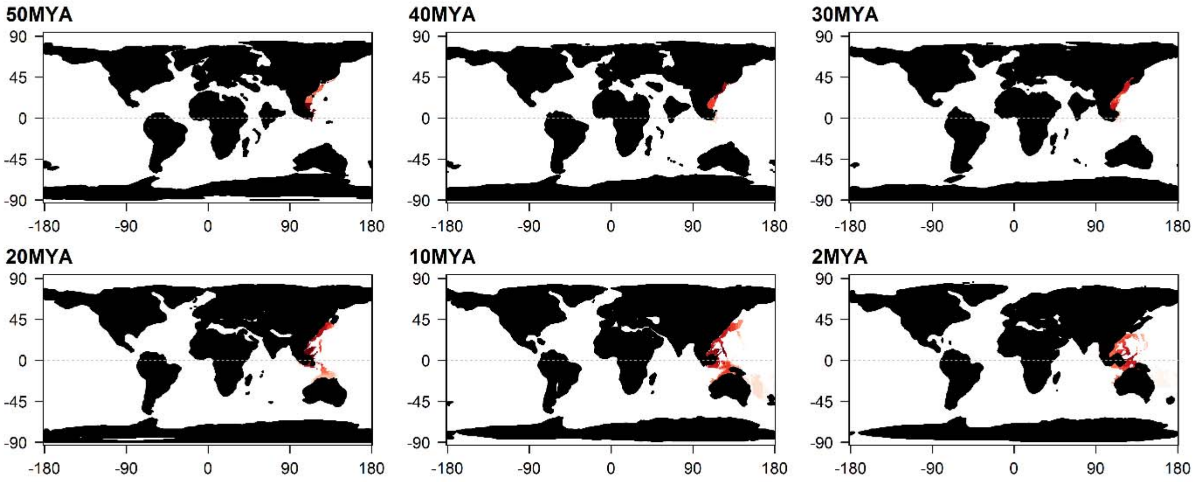

Supplementary Figure 18: Simulation with a different start. Instead of the coast of Gondwana presented in the main manuscript, the start in the simulation shown is the coast of Eastern Asia. This additional simulation with a different start east of the Asian continent suggests that other lineages, here restricted to Eastern Asia, also contributed to the biodiversity of the Indo-Australian Archipelago.

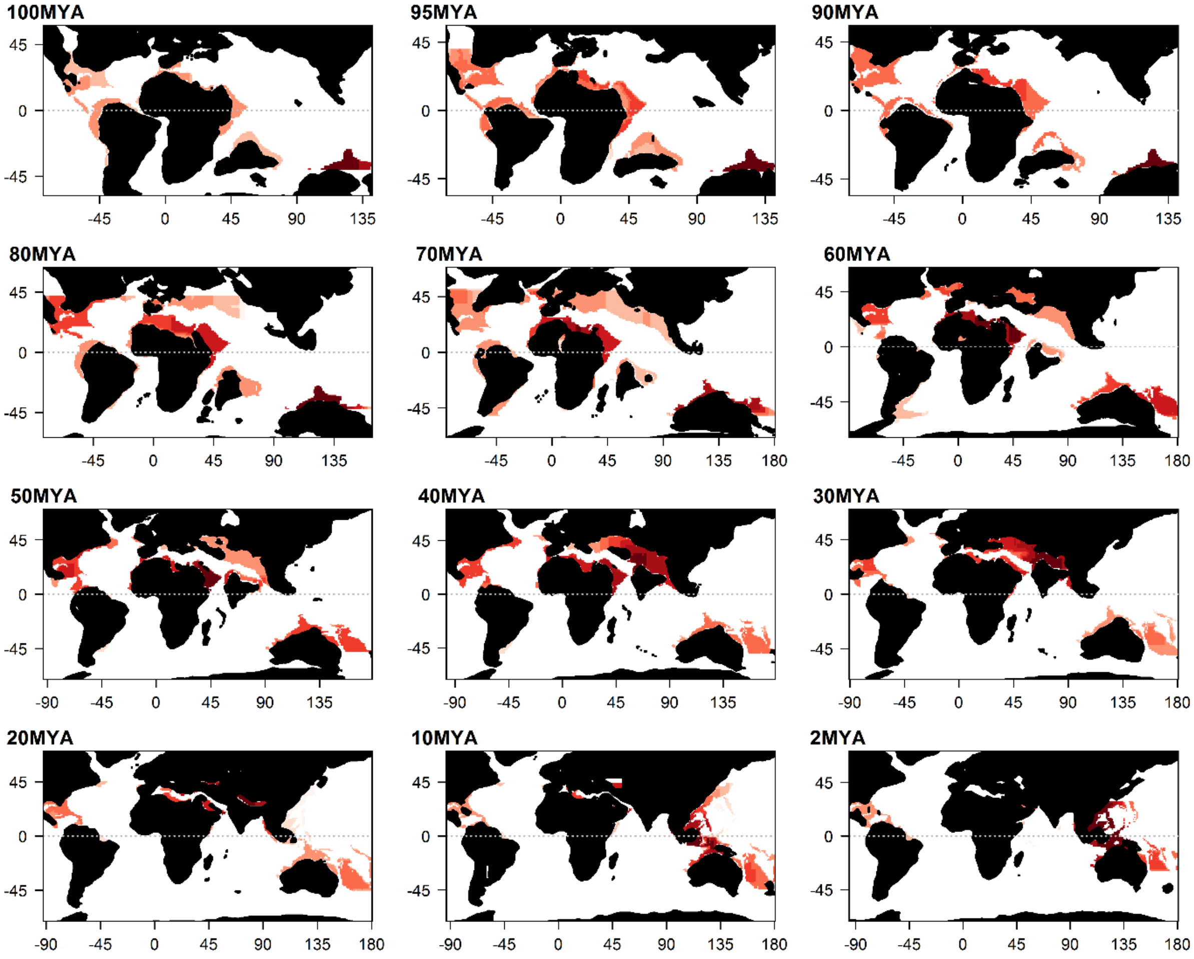

Supplementary Figure 19: Species richness through time. Results are based on the best model of sympatric speciation using parameters *d*=5 and p_s=6e-4. The colour gradient from light red to dark red represent the richness gradient from low to high rescaled between 0 and 1 for each time step. The lineages in the central and eastern Tethys had a high diversification given the extensive reef habitat in that region. As for the parapatric model, a small portion of the lineage migrated to the central Pacific, while the species in the Mediterranean region got extinct at the closure of the Mediterranean Sea. Some of the Australian fauna separated early after the northward movement of the subcontinent of India

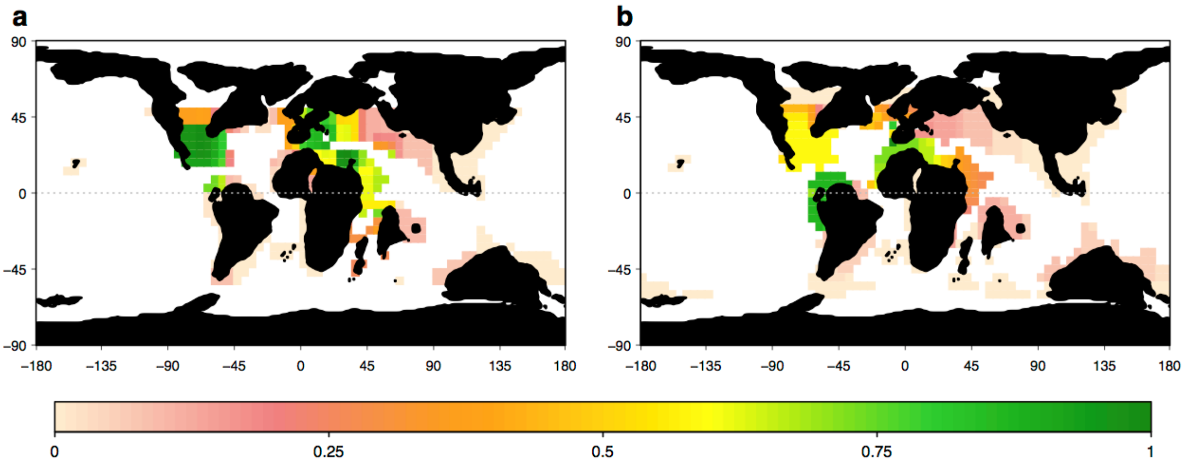

Supplementary Figure 20: Coral fossil richness. Shown are (a) results of the simulation with the parapatric model with *d*=4 and *ds*=5 and (b) observed for coral fossil richness for the late Cretaceous (70±5 Ma). This figure illustrates that the biodiversity hotspot in the west Tethys existed prior to the K/Pg transition, but also extended into the paleo-Atlantic.

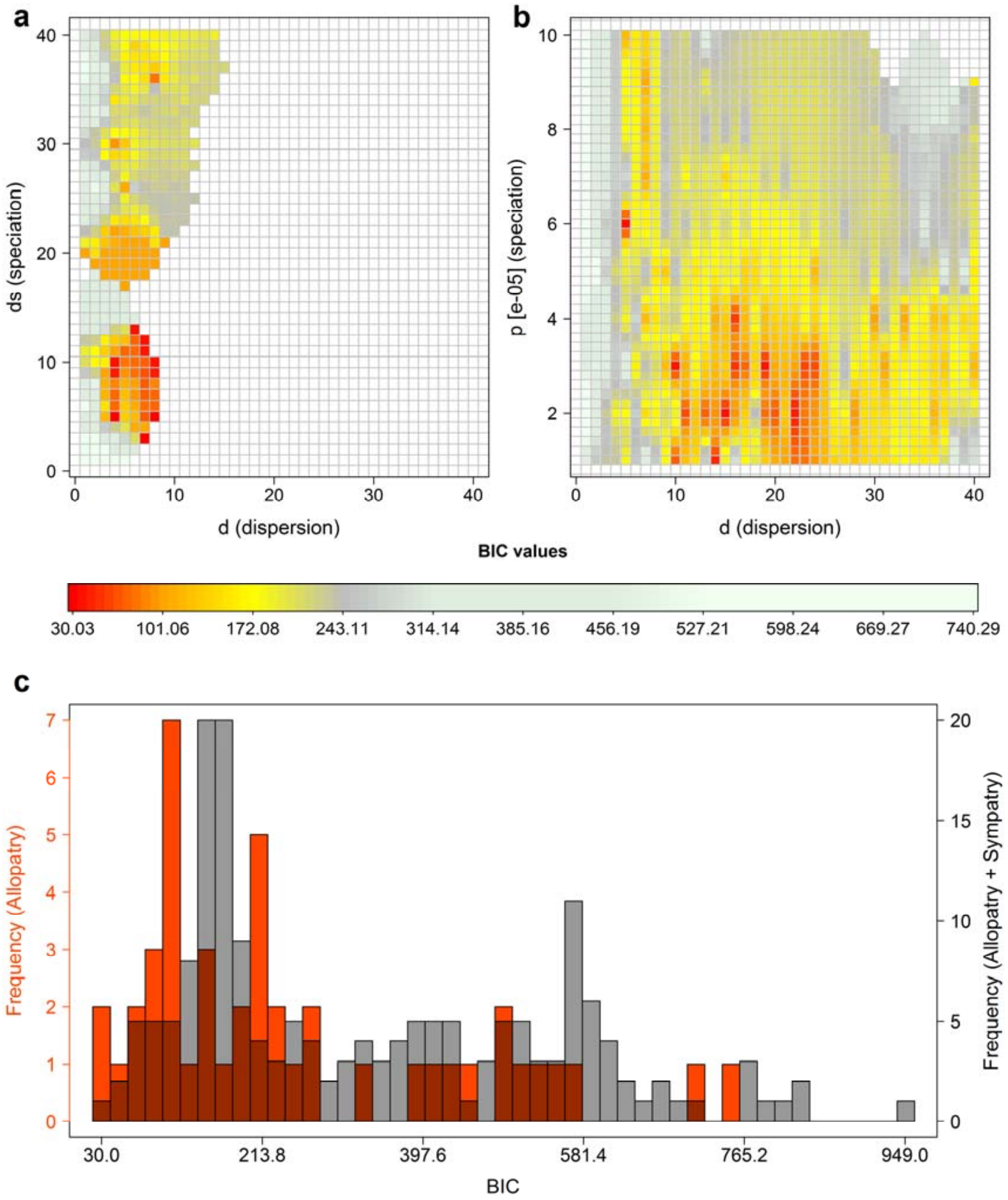

Supplementary Figure 21: Performance of the simulations. Performance was quantified with the Bayesian Information Criterion (BIC) in the explored parameter space shown for a) Parapatric speciation, b) sympatric speciation and c) the comparisons of parapatric models in red and models combining parapatric and sympatric modes of speciation. In a) and b), the colour from light blue to red represents the gradient of high to low BIC. White values represent the parameter space, where simulations generated too many species (>20’000). The best performing parapatric simulations were associated with lower values of speciation and dispersal, while the best performing sympatric simulations were more spread within the parameter space with low to intermediate dispersal

parameters. The simulations with the parapatric or the sympatric modes of speciation showed lower BIC values than the models combining both modes of speciation.

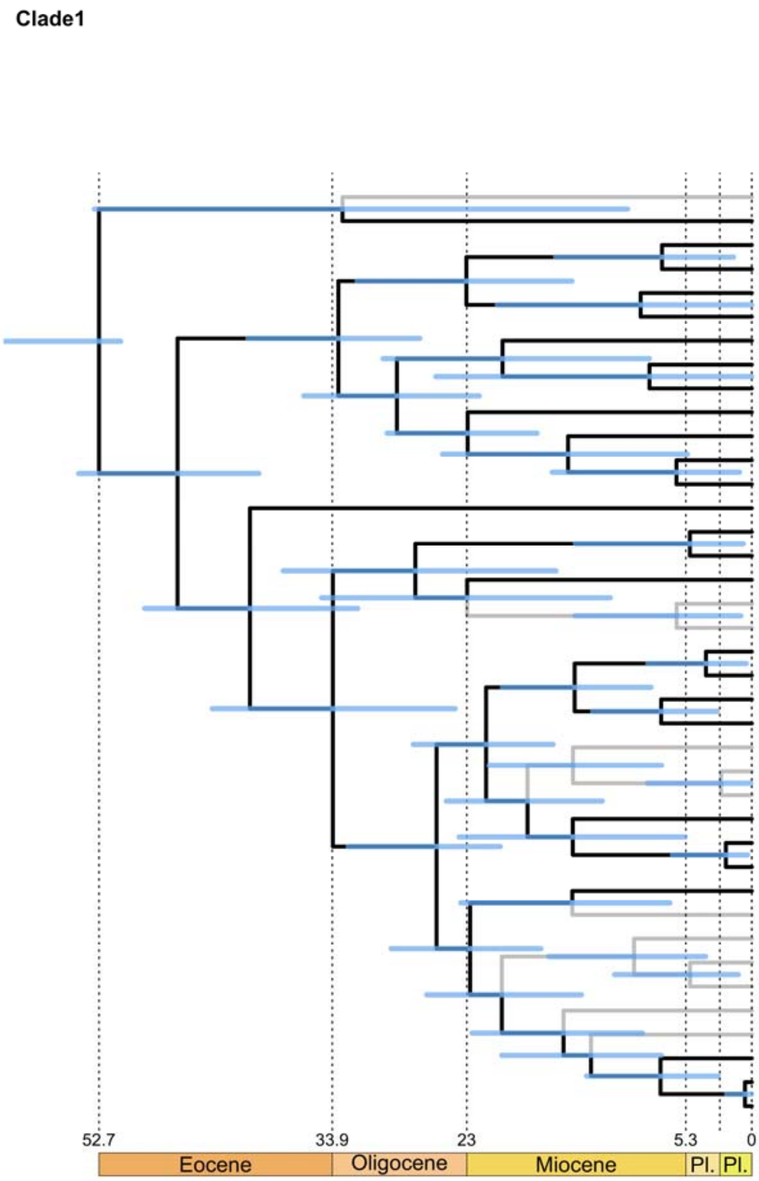

Clade2

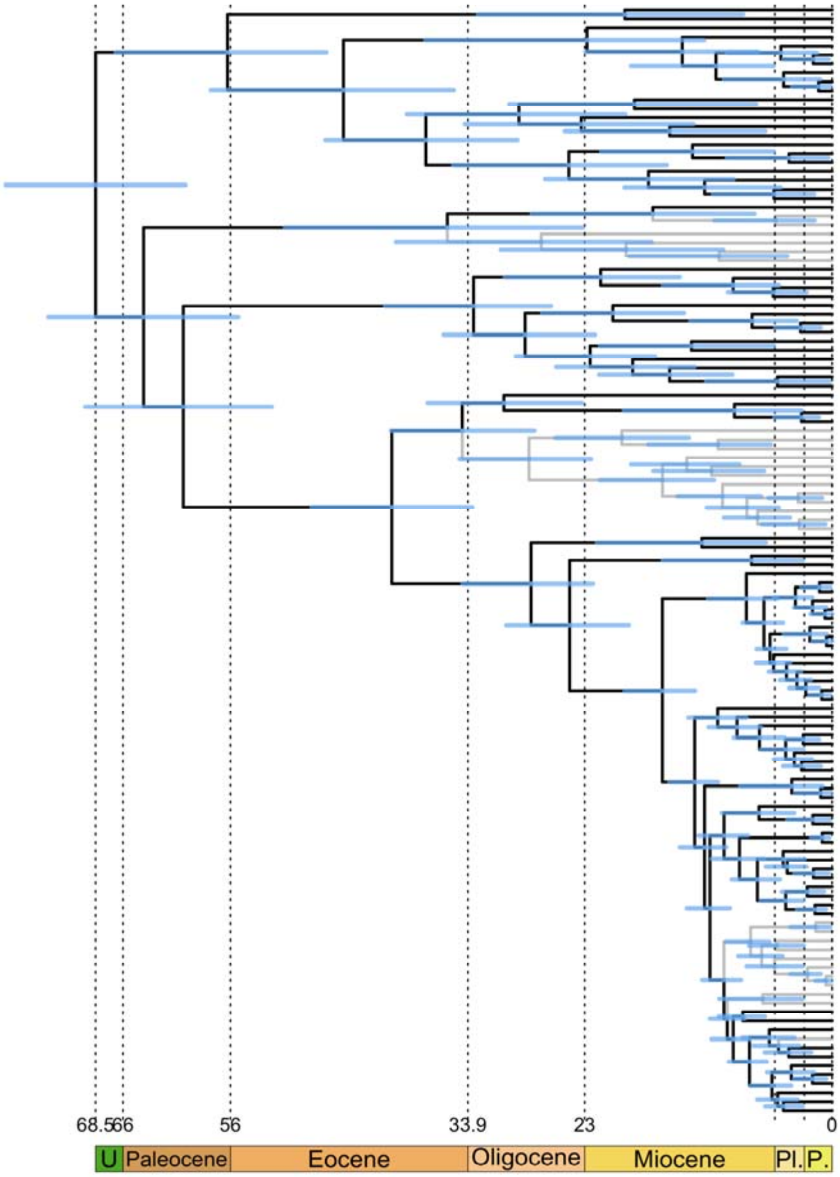

Clade3

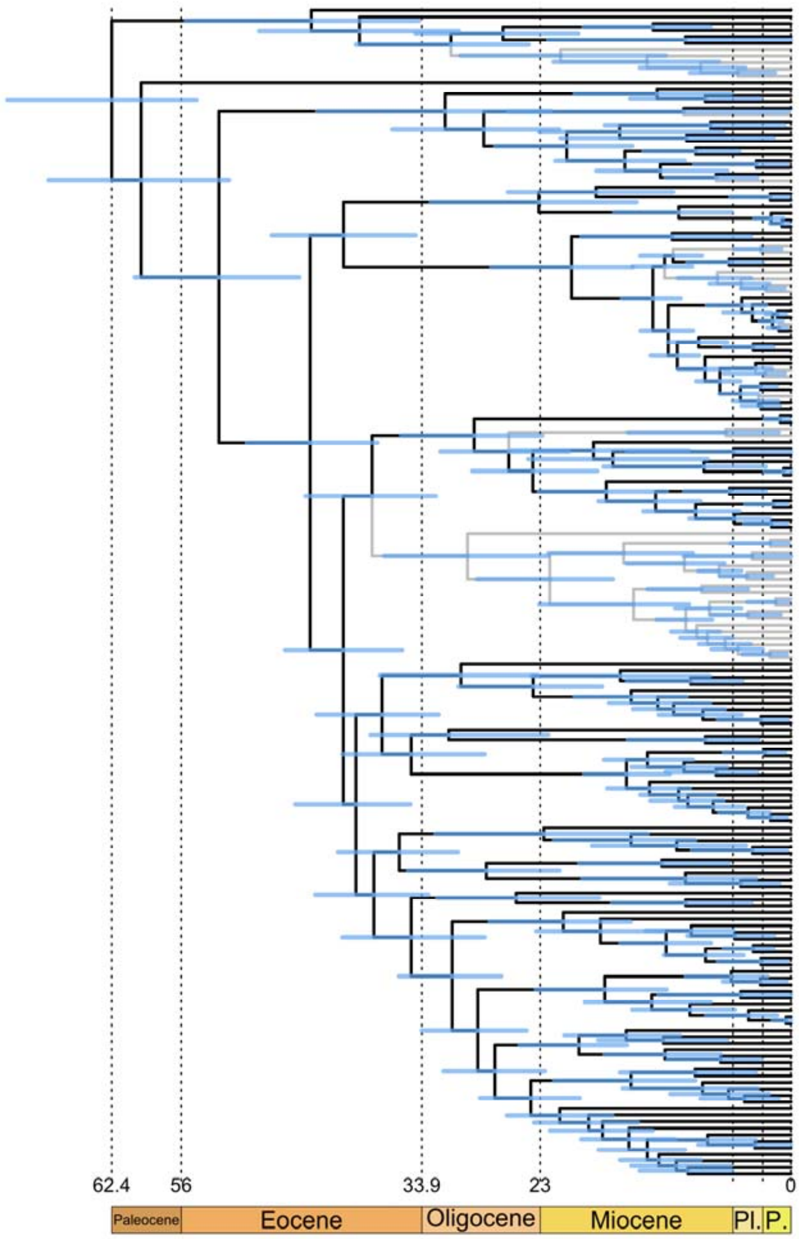

Supplementary Figure 22: Labridae phylogeny. Phylogeny of Labridae with the dating uncertainty associated to each node. The clade 1 contains the hypsigenyines lineage, the clade 2 contains the scarines, the

labrines, the cheillines and the pseudocheilines lineages, the clade 3 contains the pseudolabrines, the novaculines and the julidines lineages. The grey colour represent the Atlantic and TEP lineages, while black represent the Indo-Pacific lineages.

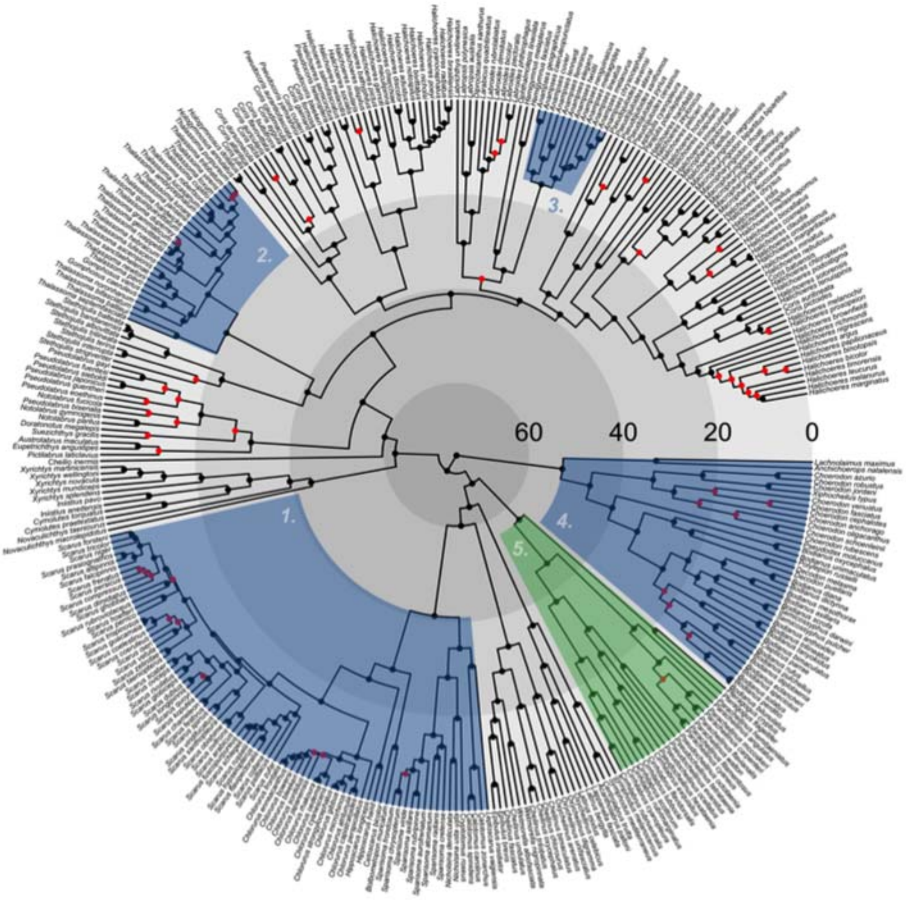

Supplementary Figure 23: Labridae phylogeny. Radial cladogram of the family Labridae showing the posterior probabilities obtained for each node with the Bayesian inference: Red dots $p<0.5$, Black dots $p>0.5$. Concentric circles are placed every 20Ma. Highlighted clades: 1. Scarines, 2. Thalassoma, 3. Anampses, 4. Hypsigenyines, 5. Pseudocheilines. The green colour indicates that this clade was found in another position in previous reconstructions.

Supplementary Table 1. Substitution models selected with Jmodeltest 2.1.6[4,5] for each marker. The models were selected using the Bayesian Information Criterion and were used as independent substitution models in MCMC estimations

**Locus**	12S	16S	COI	Cytb	RAG2	TMO-4c4	S7
**Selected Model**	TIM2+I+G	GTR+I+G	TrN+I+G	GTR+I+G	TIM2+I+G	TPM2+I+G	TPM1+I+G

Supplementary Table 2. Description of calibrations used in the estimation of divergence time for the Labridae, The prior distributions were placed on the mrca of the corresponding lineages

**Family/MRCA**	**Fossil/biogeography**	**Age (Ma)**	**Distribution**	**Prior (5-95%)**	**Source publication**
Root	K/T boundary	65	Normal	54.5–105.5	[6]
Hypsigenyines	*Phyllopharyngodon longipinnis*	50	Lognormal	51.5–63.1	[7]
Labridae(-hypsigenyines)	*Eocoris bloti* *Bellwoodilabrus landini*	50	Lognormal	51.5–63.1	[8]
Pseudodax ⁄ Achoerodus	*Trigondon jugleri*	14	Lognormal	15.1–44.0	[9]
Calotomus ⁄ Sparisoma	*Calotomus preisli*	14	Lognormal	15.1–44.0	[10]
Bolbometopon ⁄ Cetoscarus	*Bolbometopon sp.*	5	Lognormal	6.1-11.1	[10]
Halichoeres dispilus ⁄ pictus	Isthmus of Panama	3.1	Normal	3.5-10.5	[11]

Supplementary References

1. Müller, R. D., Sdrolias, M., Gaina, C. & Roest, W. R. Age, spreading rates, and spreading asymmetry of the world's ocean crust. *Geochemistry, Geophys. Geosystems* **9,** Q04006 (2008).
2. Seton, M. *et al.* Global continental and ocean basin reconstructions since 200 Ma. *Earth-Science Rev.* **113,** 212–270 (2012).
3. Torsvik, T. H. *et al.* Phanerozoic polar wander, palaeogeography and dynamics. *Earth-Science Rev.* **114,** 325–368 (2012).
4. Darriba, D., Taboada, G. L., Doallo, R. & Posada, D. jModelTest 2: more models, new heuristics and parallel computing. *Nat. Methods* **9,** 772–772 (2012).
5. Guindon, S. *et al.* New algorithms and methods to estimate maximim-likelihood phylogenies: Assessing the performance of PhyML 3.0. *Syst. Biol.* **59,** 307–321 (2010).
6. Bellwood, D. R. & Wainwright, P. C. in *Coral reef fishes: dynamics and diversity in a complex ecosystem* (ed. Press, A.) 5–32 (2002).
7. Bellwood, D. R. A new fossil fish Phyllopharyngodon longipinnis gen. et sp. nov. (family Labridae) from the Eocene, Monte Bolca, Italy. *Stud. e Ric. sui giacimenti terziari di Bolca* **6,** 149–160 (1990).
8. Bannikov, A. F. & Sorbini, L. Coris bloti, a new genus and species of labrid fish (Perciformes, Labroidei) from the Eocene of Monte Bolca. *Italy. Stud. e Ric. sui giacimenti Terziari di Bolca* **6,** 133–148 (1990).
9. Bannikov, A. F. & Carnevale, G. Bellwoodilabrus landinii n. gen., n. sp., a new genus and species of labrid fish (Teleostei, Perciformes) from the Eocene of Monte Bolca. *Geodiversitas* **32,** 201–220 (2010).
10. Bellwood, D. R. & Schultz, O. in *Annalen des Naturhistorischen Museums in Wien* **92,** 55–71 (1988).
11. Barber, P. H. & Bellwood, D. R. Biodiversity hotspots: evolutionary origins of biodiversity in

wrasses (Halichoeres: Labridae) in the Indo-Pacific and new world tropics. *Mol. Phylogenet. Evol.* **35,** 235–253 (2005).
